# Constructing a prognostic model for head and neck squamous cell carcinoma based on glucose metabolism related genes

**DOI:** 10.3389/fendo.2023.1245629

**Published:** 2023-10-09

**Authors:** Yu Liu, Nana Liu, Xue Zhou, Lingqiong Zhao, Wei Wei, Jie Hu, Zhibin Luo

**Affiliations:** ^1^ Department of Oncology, Chongqing General Hospital, Chongqing, China; ^2^ Department of Onclogy, People’s Hospital of Chongqing Hechuan, Chongqing, China; ^3^ Department of Oncology, The Affiliated Hospital of Southwest Medical University, Luzhou, China; ^4^ Department of Otolaryngology Head and Neck Surgery, Chongqing General Hospital, Chongqing, China

**Keywords:** glucose metabolism, head and neck squamous cell carcinoma, immune microenvironment, therapeutic target, drug sensitivity, prognosis

## Abstract

**Background:**

Glucose metabolism (GM) plays a crucial role in cancer cell proliferation, tumor growth, and survival. However, the identification of glucose metabolism-related genes (GMRGs) for effective prediction of prognosis in head and neck squamous cell carcinoma (HNSC) is still lacking.

**Methods:**

We conducted differential analysis between HNSC and Normal groups to identify differentially expressed genes (DEGs). Key module genes were obtained using weighted gene co-expression network analysis (WGCNA). Intersection analysis of DEGs, GMRGs, and key module genes identified GMRG-DEGs. Univariate and multivariate Cox regression analyses were performed to screen prognostic-associated genes. Independent prognostic analysis of clinical traits and risk scores was implemented using Cox regression. Gene set enrichment analysis (GSEA) was used to explore functional pathways and genes between high- and low-risk groups. Immune infiltration analysis compared immune cells between the two groups in HNSC samples. Drug prediction was performed using the Genomics of Drug Sensitivity in Cancer (GDSC) database. Quantitative real-time fluorescence PCR (qRT-PCR) validated the expression levels of prognosis-related genes in HNSC patients.

**Results:**

We identified 4973 DEGs between HNSC and Normal samples. Key gene modules, represented by black and brown module genes, were identified. Intersection analysis revealed 76 GMRG-DEGs. Five prognosis-related genes (MTHFD2, CDKN2A, TPM2, MPZ, and DNMT1) were identified. A nomogram incorporating age, lymph node status (N), and risk score was constructed for survival prediction in HNSC patients. Immune infiltration analysis showed significant differences in five immune cell types (Macrophages M0, memory B cells, Monocytes, Macrophages M2, and Dendritic resting cells) between the high- and low-risk groups. GDSC database analysis identified 53 drugs with remarkable differences between the groups, including A.443654 and AG.014699. DNMT1 and MTHFD2 were up-regulated, while MPZ was down-regulated in HNSC.

**Conclusion:**

Our study highlights the significant association of five prognosis-related genes (MTHFD2, CDKN2A, TPM2, MPZ, and DNMT1) with HNSC. These findings provide further evidence of the crucial role of GMRGs in HNSC.

## 1 Introduction

Head and neck cancer is one of the most common malignant tumors, with the sixth highest incidence in the world, and the most common pathological type is squamous cell carcinoma (1). The global annual incidence and mortality of head and neck squamous cell carcinoma (HNSC) are estimated to be 900,000 and 450,000 deaths (2, 3), respectively. Since the early symptoms of HNSC are not obvious, most HNSC patients are diagnosed at an advanced stage, with a poor prognosis and a 5-year survival rate of less than 50% (4). Therefore, further research on the molecular mechanism of HNSC and the development of effective early screening, diagnosis and treatment methods are crucial to improve the prognosis of HNSC patients.

Changes in glucose metabolism (GM) are critical to the growth and progression of cancer (5), which mainly involve four aspects: tricarboxylic acid cycle, glycolysis, gluconeogenesis and glycogen synthesis (6). Traditionally, it is believed that cancer cells metabolize glucose mainly through glycolysis to produce sufficient energy and other key metabolites needed for survival. Glucose is processed through glycolysis to produce ATP and pyruvate, and then through the pentose phosphate pathway to produce ribose 5-phosphate and NADPH, or enter the tricarboxylic acid (TCA) cycle in the mitochondria. Glucose-derived citrate is converted to acetyl-CoA, oxaloacetic acid (OAA) or a-ketoglutaric acid (a KG). Glutamine is deaminated to form glutamate, which is processed to produce a KG for use in the TCA cycle (7). This classical type of metabolic change provides the substrates required for cancer cell proliferation and division (8), which are involved in tumor growth, metastatic progression and long-term survival. Studies have shown that the number of genes related to glycolysis is associated with tumor proliferation, invasion, angiogenesis, chemotherapy and radiotherapy resistance, and there is a correlation between glycolysis and clinical outcomes (9, 10). The glucose metabolism of cancer cells is mainly regulated by a series of transcription factors, including c-Myc, p53, HIF-1α, etc, under the interaction of signaling pathways dominated by Akt, PI3K, PTEN, mTOR, and AMPK (11, 12). So far, targeted drugs targeting tumor glucose metabolism have been released, such as GLUT-1 inhibitors, LDHA inhibitors, IDH2 mutation inhibitors, etc. (13). Although many prognostic models for HNSC have been constructed by researchers, the effectiveness and sensitivity need to be improved, so it is necessary to construct more accurate prognostic models to improve the prognosis of HNSC patients (14–18). Besides, in HNSC, there is still a lack of glucose metabolism related genes (GMRGs) signature to predict patient prognosis more effectively.

Machine learning offers significant advantages and impressive progress in identifying disease biomarkers (19–22). While traditional biomarker research usually takes a lot of time and resources, machine learning methods can efficiently extract key features from large-scale biological data and accelerate the biomarker discovery process (23–25). In addition, machine learning can integrate multiple data sources, such as genomics, transcriptomics and proteomics data, to reveal the molecular mechanisms of diseases at different levels and provide more reliable biomarkers for precision medicine (26–28). Overall, the advantages of machine learning in recognizing disease biomarkers lie in its efficient feature selection capability, its classification ability to adapt to complex data structures (29, 30), and its integration and mining of data from multiple sources, and these advances have brought new hope to the fields of disease diagnosis, treatment, and prognosis assessment (31–33). However, with the continuous development of technology, there are still many challenges and opportunities waiting to be explored and solved in our understanding and application of disease biomarkers.

In this study, based on genes related to glucose metabolism, a series of bioinformatics methods such as differential expression analysis, weighted gene co-expression network analysis (WGCNA), gene set enrichment analysis (GSEA) functional enrichment and immune infiltration analysis were used to establish a prognostic model of HNSC and explore its pathogenesis.

## 2 Materials and methods

### 2.1 Data sources

Clinical information data and RNA-sequencing (RNA-seq) were acquired from The Cancer Genome Atlas (TCGA) database. There were 500 head and neck squamous cell carcinoma (HNSC) and 44 Normal samples with clinical information in the TCGA database. External validation dataset GSE65858 of Gene Expression Omnibus (GEO) database has 270 patients with survival information. In the GeneCard database, 605 GMRGs were obtained by Relevance score ≥ 2.

### 2.2 Analysis of differential expression and WGCNA

Differentially expressed genes (DEGs) between 500 HNSC and 44 Normal samples were obtained by limma (version 3.42.2) package (|log_2_FC| >0.5 and p.value < 0.05) (34). Then, volcano and heat map were drawn by ggplot2 (version 3.3.2) and pheatmap (version 1.0.12) packages (35), respectively. In this study, to find out the genes associated with different traits, 544 samples in TCGA-HNSC were used as traits for WGCNA analysis. 500 HNSC and 44 Normal samples were employed in build a co-expression network by WGCNA (version v1.70-3) package (36). Firstly, samples clustered were performed on 500 HNSC and 44 Normal samples, and outlier samples were eliminated to secure the precision of the analysis. The soft threshold was resolved to ensure that the engagement between genes conforms to the scale-free distribution to the maximum extent. The module was divided by dynamic cut tree algorithm, and the parameter minModuleSize were set to 300. The key module was acquired by correlation analysis between module and HNSC.

### 2.3 Identification of GMRG-DEGs and the prognostic risk model

DEGs between HNSC and Normal groups, genes in key module and 605 GMRGs were crossed to identify GMRG-DEGs. In addition, to assess whether GMRG-DEGs were significantly different from the survival of HNSC patients, we extracted GMRG-DEGs expression data from TCGA-HNSC samples expression data. Then, the TCGA-HNSC samples were stochastic divided into training set and test set in a ratio of 7: 3 (37). Furthermore, univariate and multivariate Cox regression analyses were performed in the training set to verify whether these genes were risk factors. Next, univariate Cox regression analysis was conducted on GMRG-DEGs expression profiles of the training set. Variables acquired by univariate Cox analysis were embraced in multivariate Cox analysis, followed by stepwise regression function (step). Moreover, we used the surviminer (0.4.6) package to calculate the cut-off value of continuous independent variables of survival data (train: 1.28; test: 1; GSE65858: 3.17), HNSC samples were separated into high-and low-risk groups based on cut-off values. For each patient, the risk score was calculated by combining the expression levels of these genes with their corresponding coefficients: Risk score = ExpressionmRNA1 × CoefmRNA1 + ExpressionmRNA2 × CoefmRNA2 + ExpressionmRNAn × CoefmRNAn. Based on the two groups, Kaplan-Meier (K-M) curve were plotted. Finally, to further assessment the effectiveness of the risk model, we plotted receiver operating characteristic (ROC) curves with 1-, 3- and 5-years as survival time nodes according to the risk model obtained by multivariate risk regression. After constructing the risk model, it was checked by TCGA test set and GSE65858 external verification set in turn. Subsequently, the risk curve, K-M curve of two groups and ROC curves were drawn for validation data.

### 2.4 Independent prognostic risk model

To further study the clinicopathological features and prognosis of risk model, in the risk model, gender, stage, age, grade and TMN stage were included. Univariate and multivariate Cox independent prognostic analysis were performed using the survival (3.2-7) package (38–40). Then, based on the TCGA-HNSC training set of 316 samples with clinical information, a nomogram was constructed using rms (version 6.2-0) package to project the 1-, 3- and 5-year survival rate for HNSC patients. Moreover, calibration curves were drawn to evaluate the precise of the prediction.

### 2.5 Functional enrichment and immune microenvironment analyses

GSEA on all genes in two groups were performed by GSEA software (v4.1.0). SIZE > 20 and NOM.p.val < 0.05 were set as significantly enriched pathways. Furthermore, to study immune cell infiltration in two groups, Cell type Identification By Estimating Relative Subsets Of RNA Transcripts (CIBERSORT) algorithm and LM22 gene set were used to calculate the proportion of 22 immune cells of all HNSC samples (high-risk = 96, low-risk = 254) in two groups, and excluding samples with p > 0.05 (remaining samples high-risk = 95, low-risk = 252). Then, according to the score of each immune cell in two groups, the score heat map of 22 immune cells was drawn. Differences in immune cells between two groups were compared by Wilcoxon test, and ggplot2 package was employed to draw violin plot. Subsequently, Spearman correlation analysis of immune cells and prognostic related genes were conducted. Immune score and matrix score of the TCGA-HNSC transcriptome data were calculated.

### 2.6 Immunotherapy responsiveness analysis and sensitivity analysis of chemical drugs

Firstly, we calculated tumor mutation burden (TMB) and microsatellite instability (MSI) for two groups. Then, Dysfunction, Exclusion and tumor immune dysfunction and exclusion (TIDE) for the two groups were estimated and analyzed. In addition, Pearson correlation analysis were performed on prognostic related genes and TIDE (39). Finally, according to the above HNSC samples in two groups of the Genomics of Drug Sensitivity in Cancer (GDSC) database, a ridge regression model was constructed to predict the half maximal inhibitory concentration (IC50) values of drugs by pRRophetic algorithm. Moreover, calculation of drug level of expression in two groups was performed by Wilcoxon test.

### 2.7 qPCR assay

HNSC tumor and paracancerous tissue samples were obtained from HNSC patients with knowledge and consent from Chongqing general hospital, and this study was approved by the Chongqing general hospital ethics committee. Seven pairs of frozen tissue samples were divided into two groups, of which seven paracancerous tissue samples were Normal group and the other seven tumor tissue samples were HNSC group (Case). Then, total RNA of samples was isolated and purified by TRIzol (Ambion) reagent following the instruction manual. Then, the extracted RNA was tested for concentration by NanoPhotometer N50. Next, reverse transcription *via* SureScript-First-strand-cDNA-synthesis-kit (Servicebio) by an ordinary PCR instrument. Reverse transcription product cDNA was diluted 5-20 times with ddH_2_O (RNase/DNase free). Subsequently, polymerase chain reaction (PCR) amplification reaction was performed by CFX96 real-time quantitative PCR instrument. 1 min at 95 °C (pre-denaturation), followed by at 95 °C for 20 s (denaturation), 55 °C for 20 s (annealing) and 72 °C for 30 s (elongation). The above reactions were subjected to forty cycles. Primer sequences were showed in **Table 1**.

**Table 1:** Primer sequences used in the quantitative reverse transcriptase PCR (qRT-PCR).

primer	sequence
DNMT1 F	GAGGAGGGCTACCTGGCTAA
DNMT1 R	CGGGCTTCACTTCTTGCTTG
MPZ F	ATGCCATTTCGATCTTCCACT
MPZ R	GAGGTCTTGCCCACTATGTCTG
TPM2 F	TCACCAGACCTTGGACCAGA
TPM2 R	AGGATTAAAGGGCCTTGAGAGG
CDKN2A F	GCTAGACACAAAGGACTCGGT
CDKN2A R	CTCTGACGCGACATCTGGAC
MTHFD2 F	GGCAGTTCGAAATGAAGCTGTTG
MTHFD2 R	AGGATCACACTCAGGTGTGGC
internal reference-GAPDH F	CGAAGGTGGAGTCAACGGATTT
internal reference-GAPDH R	ATGGGTGGAATCATATTGGAAC

### 2.8 Statistical analysis

The qPCR results were analyzed using GraphPad Prism Software (version 8.3.0). The data are presented as means ± standard deviation (SD) from three independent experiments and were analyzed by analysis of variance (ANOVA). A p-value less than 0.05 was considered statistically significant.

## 3 Results

### 3.1 Acquisition of DEGs and key module genes

**Figure 1:**
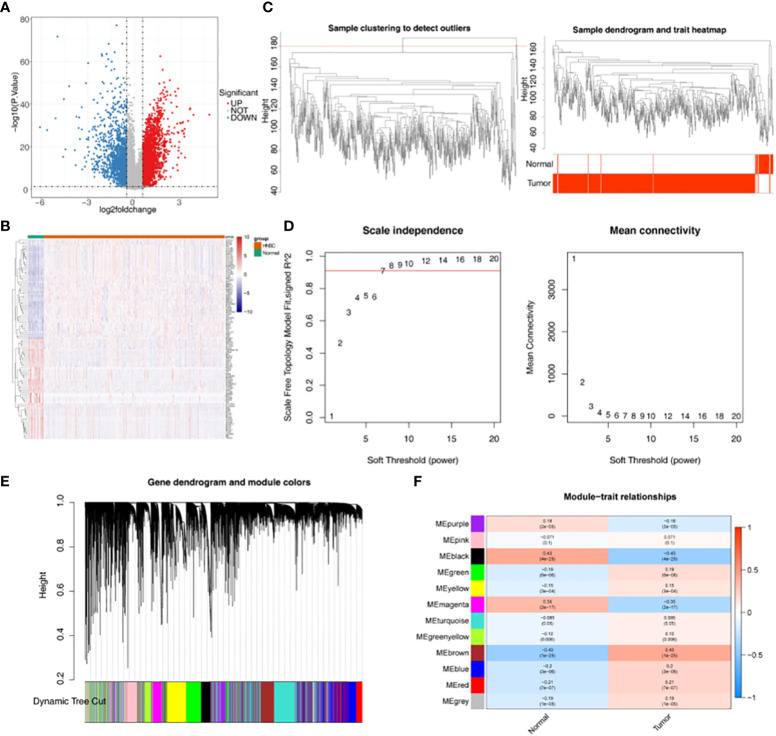
Acquisition of differentially expressed genes (DEGs) and key module genes. **(A)** Volcano plot of DEGs between HNSC and Normal samples (|log2FC| >0.5 and p.value < 0.05). **(B)** Heatmap of Top100 DEGs between HNSC and Normal samples. **(C)** Sample clustering dendrogram to remove outliers and trait heatmap. **(D)** Analysis of the scale-free fit index (left) and the mean connectivity (right) for various soft-thresholding powers (Soft threshold = 7). **(E)** Cluster dendrogram of all DEGs under the clustering tree. **(F)** Correlations heatmap between modules and traits.

There were 4973 DEGs between HNSC and Normal groups (**Figure 1A**). Heat map showed the expression of up-regulated and down-regulated top 100 DEGs between HNSC and Normal groups (**Figure 1B**). Samples clustering result indicated there were 5 outlier samples. Therefore, the remaining of 539 samples were used for subsequent analysis (**Figure 1C**). When the soft threshold is 7, the network was closest to the distribution without network scale (**Figure 1D**). 12 modules were obtained by dynamic cut tree algorithm (**Figure 1E**). Black module gene (1590 genes) and brown module gene (3487 genes) were selected as key modules (**Figure 1F**).

### 3.2 Acquisition of GMRG-DEGs and the evaluation of prognostic risk model

**Figure 2:**
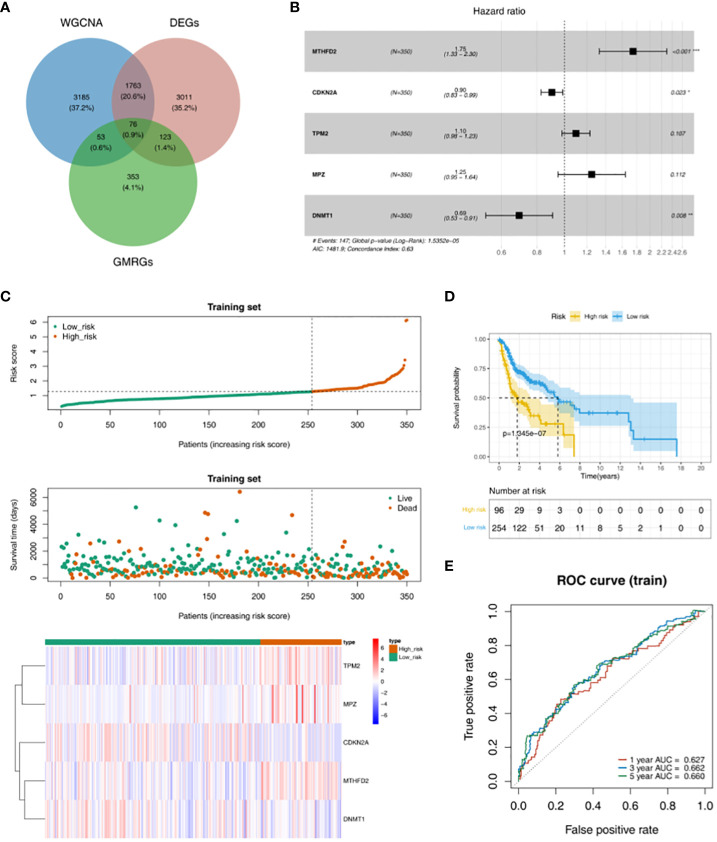
Obtaining GMRG-DEGs and evaluation of the prognostic risk model in training set. **(A)** Venn diagram of module genes, DEGs and glucose metabolism related genes (GMRGs) for screening GMRG-DEGs. **(B)** Forest plot of multivariate Cox regression analysis. **(C)** Distribution of risk scores, survival times gene expressions of high and low risk groups in the training set. **(D)** Survival curve of high- and low-risk groups in the training set. **(E)** ROC curves of 1-, 3-, and 5-year based on the training set.

According to the intersection of DEGs between HNSC and Normal groups, genes in key module and 605 GMRGs, there were 76 GMRG-DEGs (**Figure 2A**, **Table S1**). 8 prognosis related genes (TP73, TXNDC9, MTHFD2, CDKN2A, TPM2, MPZ, DNMT1 and IGF2BP2) were obtained by univariate Cox regression analysis (**Table 2**). There were 5 prognosis related genes (MTHFD2, CDKN2A, TPM2, MPZ and DNMT1) based on multivariate Cox analysis (**Figure 2B**, **Table 3**). In the high-risk group, TPM2, MPZ and MTHFD2 were highly expressed, moreover, it was found that CDKN2A and DNMT1 had a higher expressed in low-risk group (**Figure 2C**). In two groups, there was a significant difference in the survival of HNSC patients between (*p <* 0.05), and in the high-risk group, it was found that the survival rate of HNSC patients was lower (**Figure 2D**). The area under curve (AUC) values of ROC curve were greater than 0.6, indicated that the risk model had better performance (**Figure 2E**).

**Table 2:** Univariable Cox regression analysis results.

id	HR	HR.95L	HR.95H	Pvalue
TP73	0.74016	0.60627	0.903618	0.003122
TXNDC9	1.663991	1.157881	2.391323	0.005918
MTHFD2	1.401012	1.071705	1.831508	0.013643
CDKN2A	0.90169	0.82885	0.980931	0.016042
TPM2	1.131322	1.019291	1.255666	0.02039
MPZ	1.279329	1.014195	1.613775	0.037625
DNMT1	0.789719	0.629614	0.990537	0.041131
IGF2BP2	1.152585	1.002501	1.325137	0.046037

**Table 3:** Multivariate Cox regression analysis results.

id	coef	HR	HR.95L	HR.95H	Pvalue
MTHFD2	0.557097	1.745598	1.327335	2.295662	6.72E-05
CDKN2A	-0.10193	0.903092	0.826861	0.986351	0.023488
TPM2	0.093444	1.097949	0.98012	1.229944	0.106682
MPZ	0.221411	1.247837	0.949366	1.640144	0.112417
DNMT1	-0.36682	0.692933	0.528348	0.908787	0.008019

### 3.3 Verification of the prognostic risk model

**Figure 3:**
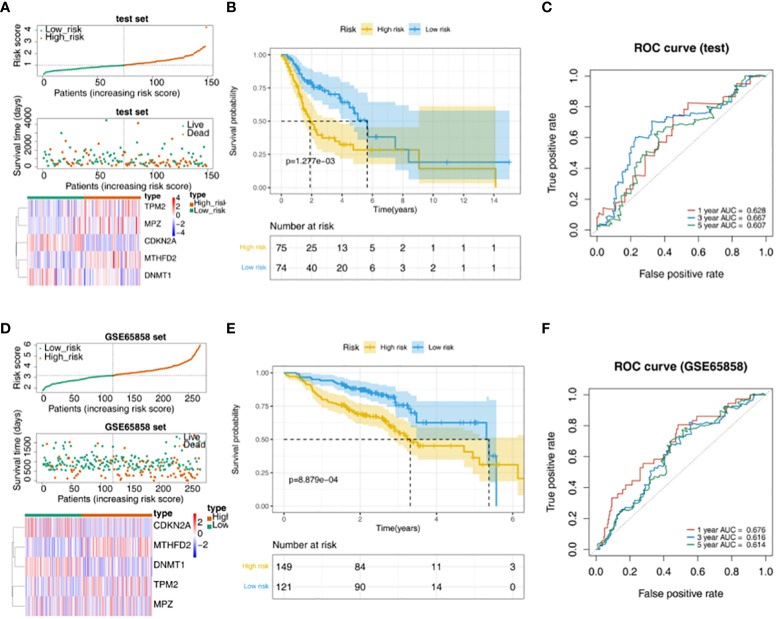
Validation of the risk model. **(A)** Distribution of risk scores, survival times gene expressions of high and low risk groups in the test set. **(B)** The survival curve of the high- and low-risk groups in the test set. **(C)** The ROC curve of 1-, 3-, and 5-year in the test set. **(D)** Distribution of risk scores, survival times gene expressions of high and low risk groups in GSE65858. **(E)** Survival curves for high- and low-risk groups in the GSE65858 dataset. **(F)** ROC curves for 1-, 3-, 5-year in the GSE65858 dataset.

TPM2, MPZ and MTHFD2 were highly expressed in the high-risk group of the TCGA test set, moreover, CDKN2A and DNMT1 had a higher expressed in the low-risk group (**Figure 3A**). The survival rate of the high-risk group in the validation data was lower (**Figure 3B**). AUC values were basically greater than 0.6, the result was basically consistent with the training set (**Figure 3C**). In addition, in the GSE65858 external verification set, it was found that TPM2, MPZ and MTHFD2 had a higher expressed in high-risk group, and we could see CDKN2A and DNMT1 had a higher expressed in the low-risk group (**Figure 3D**). In the GSE65858 dataset, K-M curve showed the survival rate was lower of the high-risk group (**Figure 3E**). Besides, it was found that the risk model was credible (**Figure 3F**).

### 3.4 Risk model evaluation of independent prognostic

**Figure 4:**
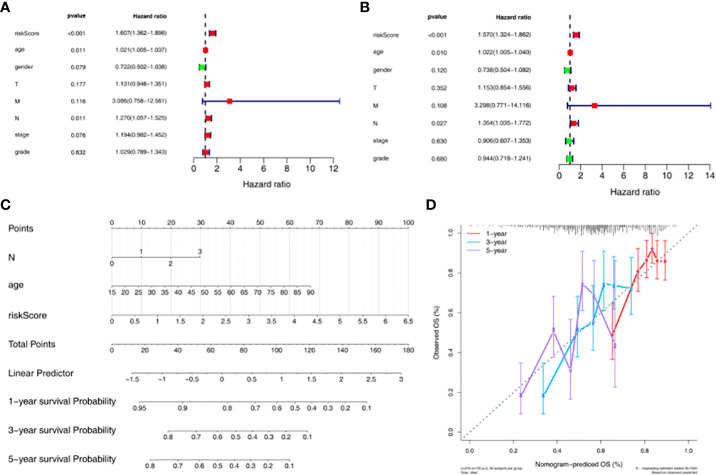
Construction and evaluation of nomogram based on independent prognostic risk models. **(A)** Univariate Cox analysis based on the training set. **(B)** Multivariate Cox analysis based on the training set. **(C)** Nomogram predicting 1-, 3-, and 5-year survival of HNSC patients. **(D)** Nomogram calibration curve.

In addition, univariate Cox independent prognostic results showed that the p.value for age, N, and riskScore was less than 0.05 (**Figure 4A**). According to the multivariate Cox independent prognostic analysis, it was found that the p.value for age, N, and riskScore were all less than 0.05. Therefore, age, N, and riskScore were considered as independent prognostic factors (**Figure 4B**). A nomogram for survival prediction in HNSC patients was constructed using age, N, and riskScore (**Figure 4C**). The calibration curve was plotted based on the above nomogram, and 3-year slope closest to 1 indicated that the prediction effect of the model could be used as an effective model (**Figure 4D**).

### 3.5 GSEA functional enrichment and immune microenvironment analyses between two groups

**Figure 5:**
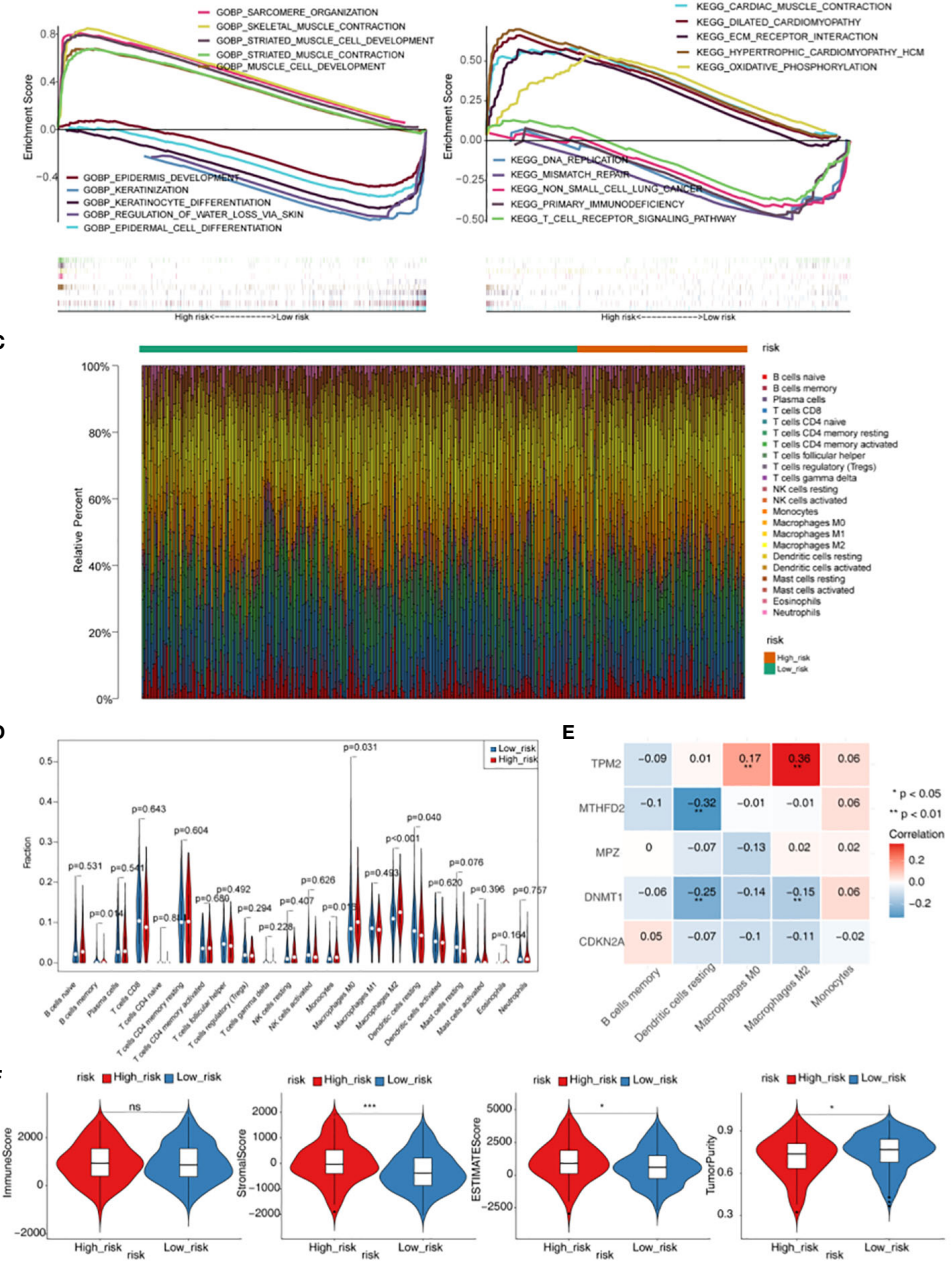
Gene set enrichment analysis (GSEA) and immune microenvironment analysis. **(A)** GO enrichment analysis of high- and low-risk groups. **(B)** KEGG enrichment analysis of high- and low-risk groups. **(C)** Heatmap of scores of 22 immune cell types in high- and low-risk groups. **(D)** Violin plot for the infiltration abundances of 22 immune cell between the high- and low-risk groups. **(E)** Heatmap of correlations between differential immune cells and five prognostic genes in the prognostic risk model. **(F)** Difference of immune score, stromal score, ESTIMATE score and tumor purity between high- and low- risk groups. * *p <* 0.05,*** *p <* 0.001. "ns" means no significance.

GO functional enrichment analysis showed that genes in high-risk groups were mainly enriched in striated muscle cell development, sarcomere organization and muscle cell development, and in low-risk groups, genes were participated in epidermis development, keratinocyte differentiation and regulation of water loss *via* skin (**Figure 5A**). In the KEGG functional enrichment analysis, it was found that genes in high-risk groups were associated with oxidative phosphorylation, cardiac muscle contraction and ecm receptor interaction. Besides, genes in low-risk groups were involved in primary immunodeficiency, DNA replication and T cell receptor signaling pathway (**Figure 5B**). Each immune cell of scores in two groups were displayed in the heat map (**Figure 5C**). The result showed that there were 5 kinds of immune cells with significant difference (*p <* 0.05), including memory B cells, Macrophages M0, Macrophages M2, Monocytes and Dendritic resting cells (**Figure 5D**). TPM2 was positively associated with Macrophages M0 and Macrophages M2, and we also could see a remarkable negative correlation between MTHFD2 and Dendritic resting cells. In addition, it was found that DNMT1 had a significantly negative associated with Macrophages M2 and Dendritic resting cells (**Figure 5E**, **Table S2**). Moreover, we could found there were significant differences in Stromal, ESTIMATE and TumorPurity scores between high- and low-risk groups (*p <* 0.05) (**Figure 5F**, **Table S3**).

### 3.6 Immunotherapeutic response and chemosensitivity analysis

**Figure 6:**
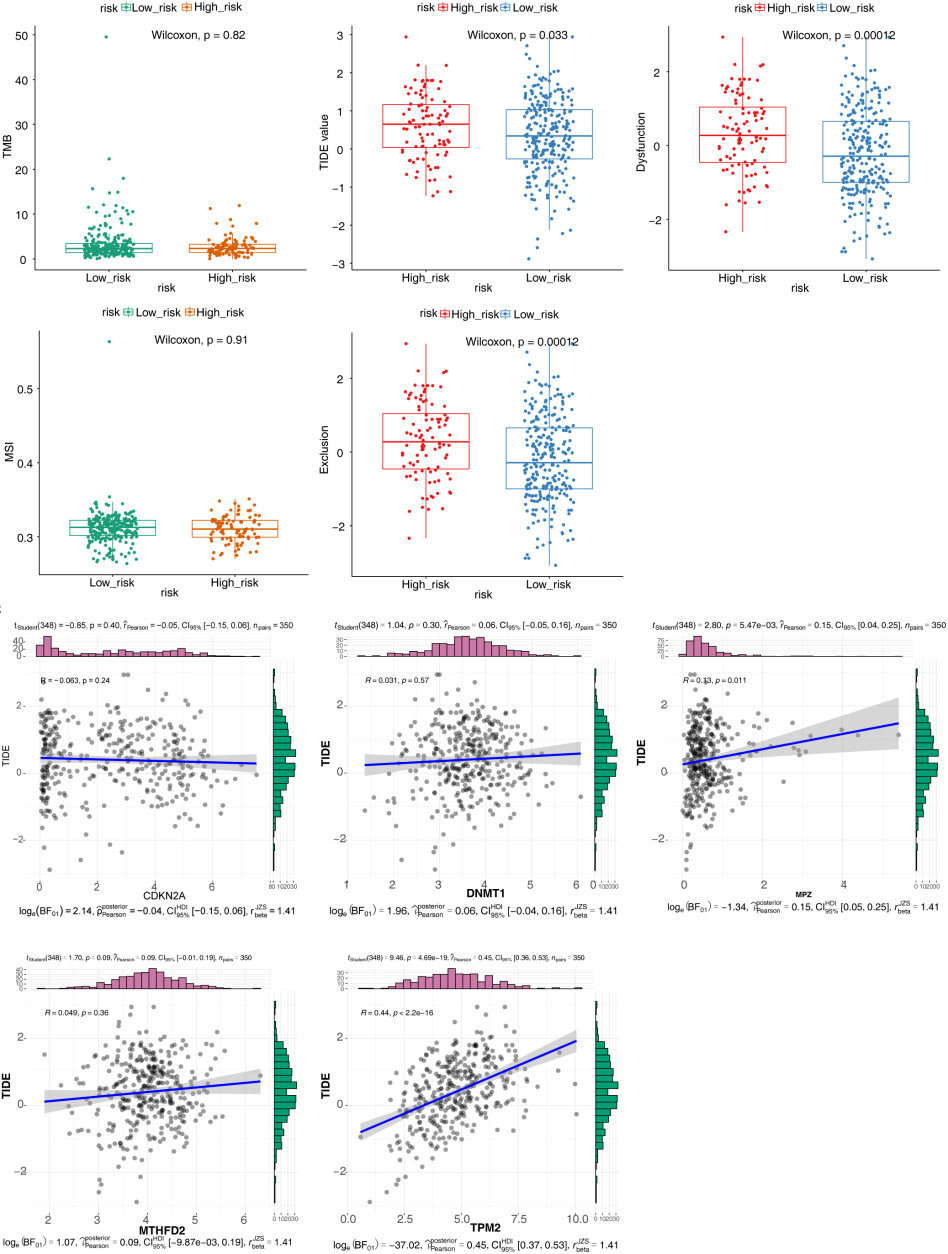
Immunotherapy responsiveness analysis. **(A)** Box plots for differences of the TMB and MSI values in high- and low-risk groups. **(B)** Box plots for differences of TIDE values, Dysfunction values and Exclusion scores in high- and low-risk groups. **(C)** Correlation scatter plots of TIDE scores with different prognostic risk model genes.

**Figure 7:**
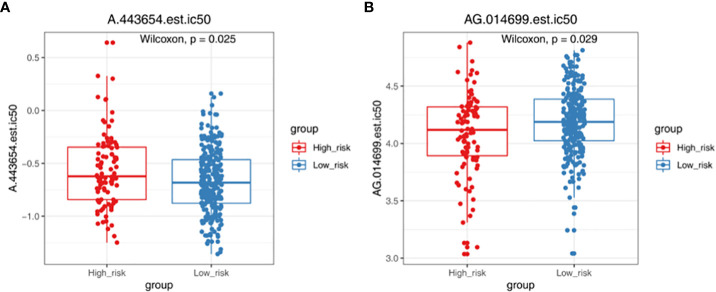
Chemotherapy responsiveness analysis. Box plots demonstrating the half maximal inhibitory concentration (IC_50_) values of A.443654 **(A)** and AG.014699 **(B)** in high- and low-risk groups.

TMB and MSI were not significant difference in two groups (**Figure 6A**). The result showed that TIDE, Dysfunction and Exclusion were remarkable differential expression in two groups (*p <* 0.05) (**Figure 6B**). It was found that TPM2 and MPZ had a certain correlation with TIDE (**Figure 6C**). There were 53 drugs significantly different between two groups, such as A.443654 and AG.014699 (**Figure 7**, **Supplementary Figure 1**, **Table S4**).

### 3.7 QuantitativeReal-timePCR (qPCR) identification

**Figure 8:**
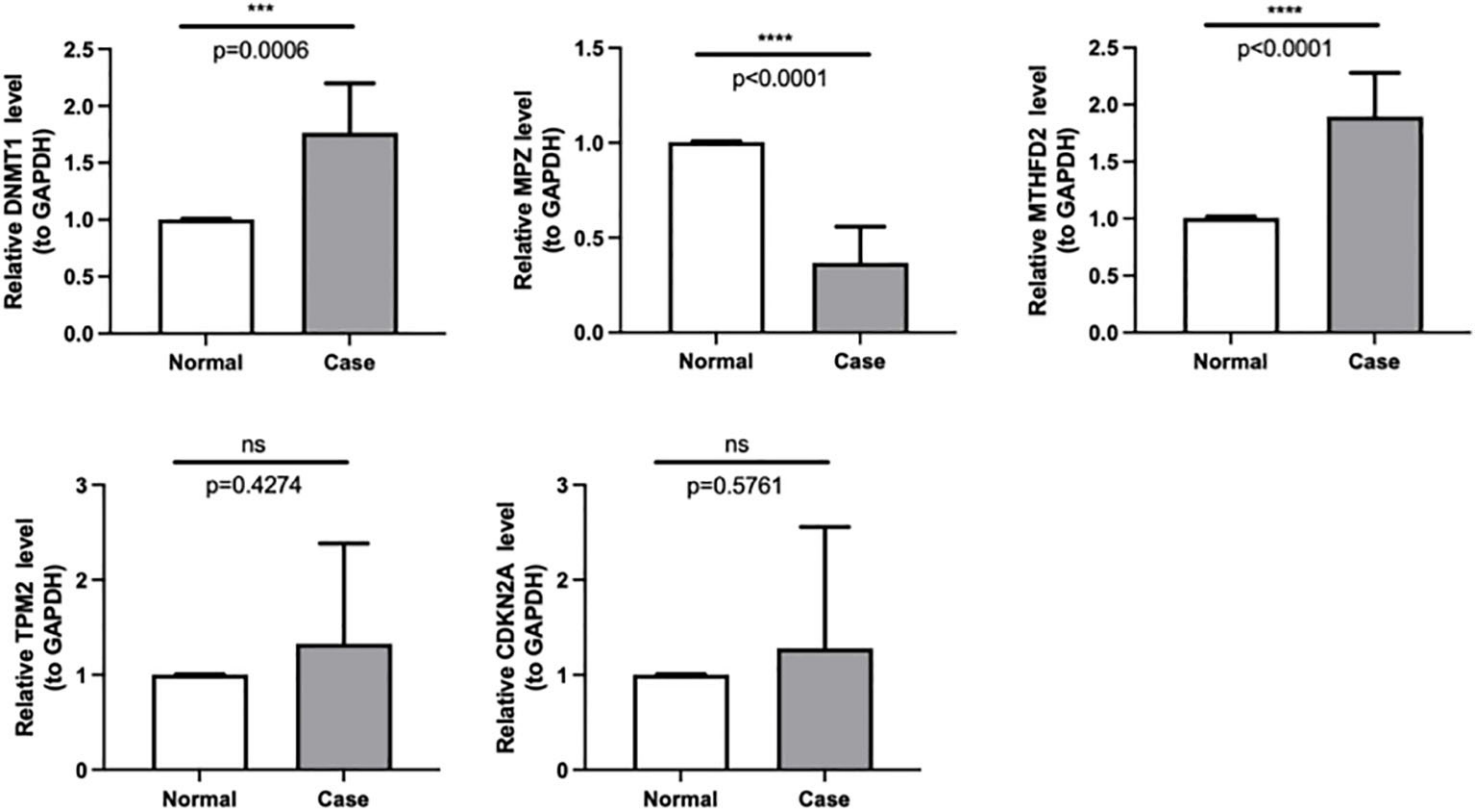
The expressions of DNMT1, MPZ, TPM2, CDKN2A and MTHFD2 were identified by quantitative real-time PCR in case and normal samples. *** *p <* 0.001,**** *p <* 0.0001. "ns" means no significance.

Based on the qPCR verification results, it can be seen that DNMT1 and MTHFD2 were up-regulated in Case (HNSC). Besides, we could see that MPZ was down-regulated in Case, and the validation results are consistent with the above analysis (**Figure 8**, **Table 4**).

**Table 4:** Results for the expressions of DNMT1, MPZ, TPM2, CDKN2A and MTHFD2 in qRT-PCR.

	Normal	Case	t, df	p.value
DNMT1	1.00041 ± 0.0046	1.7665 ± 0.4332	t=1.427 df=12	0.0006
MPZ	1.0041 ± 0.0034	0.3686 ± 0.1900	t=16.78 df=6	<0.0001
TPM2	1.0038 ± 0.0027	1.3295 ± 1.0535	t=6.786 df=11	0.4274
CDKN2A	1.0045 ± 0.0037	1.2807 ± 1.2780	t=10.92 df=11	0.5761
MTHFD2	1.0064 ± 0.0123	1.8973 ± 0.3829	t=1.907 df=12	<0.0001

## 4 Discussion

In recent years, the study of abnormal metabolism of cancer cells has become the focus of attention and is considered to be a promising area for cancer therapy. Cancer cells are characterized by consuming glucose through Warburg metabolism (41), to provide energy for their growth and proliferation. Abnormal glucose metabolism is the most prominent feature of tumor metabolism (42, 43). A study has shown that HNSC is a highly glycolytic tumor (44), and abnormal glucose metabolism is an important biological factor for the diagnosis and treatment of HNSC (45). A previous study confirmed that GMRGs such as PKM2 (pyruvate kinase) and PGK1 (phosphoglycerate kinase 1) were up-regulated in gastric cancer cell lines (46). So far, unfortunately, the role of glucose metabolism-related genes in HNSC is unclear.

In order to clarify the prognostic biomarkers related to glucose metabolism in HNSC, this study screened 605 GMRGs based on the existing HNSC gene data, and finally identified 5 key prognostic genes that may be prognostic markers or potential therapeutic targets in HNSC (MTHFD2, CDKN2A, TPM2, MPZ, and DNMT1). The mechanism of action of these five genes in tumors is summarized as follows. Methylenetetrahydrofolate dehydrogenase 2 (MTHFD2) predominantly localizes within the mitochondria and efficiently drives the folate cycle in embryonic tissues to sustain cellular proliferation. Conversely, in most adult tissues, MTHFD2 exhibits minimal to negligible expression. This enzyme possesses both dehydrogenase and cyclohydrolase activities (47). It catalyzes the conversion of CH2-tetrahydrofolic acid (CH2-THF) into 10 CHO-THF, while simultaneously converting oxidized nicotinamide adenine dinucleotide phosphate [nicotinamide adenine dinucleotide phosphate, NAD(P)+] into NAD(P)H, thus facilitating one-carbon metabolic reactions (48). It catalyzes the conversion of CH2-tetrahydrofolic acid (CH2-THF) into 10 CHO-THF, while simultaneously converting oxidized nicotinamide adenine dinucleotide phosphate [nicotinamide adenine dinucleotide phosphate, NAD(P)+] into NAD(P)H, thus facilitating one-carbon metabolic reactions (49). Studies have revealed its reactivation in diverse tumor types, and this phenomenon correlates with adverse patient prognoses (50–56). Cyclin-dependent kinase inhibitor 2A (CDKN2A), an essential tumor suppressor gene, localizes to the 21 region of human chromosome 9 (57). As a member of the cell cycle-dependent kinase inhibitor gene family, CDKN2A directly regulates the cell cycle, thus controlling cell proliferation and division. It serves as a critical tumor suppressor in various human malignancies, including colorectal cancer, exerting its preventive role by inducing cell growth arrest and senescence (58). However, homozygous deletion of CDKN2A is observed in 50% of human tumor cell lines. Its inactivation leads to malignant cell proliferation and the development of malignant tumors. Differential expression of the CDKN2A gene is evident in a variety of tumor tissues, with abnormal levels observed in tumor patients. Moreover, CDKN2A expression correlates with clinicopathological characteristics and patient prognosis (59). Tropomyosins (TPM) are actin-binding proteins that are expressed in all eukaryotes, and vertebrates have Four TPM genes containing TPM1, TPM2, TPM3, and TPM4. Recently, a large cohort study has identified TPM2 as a prognostic marker for colorectal cancer things. In colorectal cancer cell lines, the study found that TPM2 down-regulation can promote tumor proliferation and migration, whereas TPM2 over-expression attenuates the malignant phenotype of tumor cells (60). MPZ is a transmembrane protein consisting of 219 amino acids, which is a member of the immunoglobulin gene super family and has a single extracellular, transmembrane, and cytoplasmic domain (61). Recent studies have shown that MPZ is involved in the development of cancer development, report that the six cores of the MPZ are most likely to detect most clinically significant cancers but also detect many insignificant cancers (62). Most of the articles in MPZ are in the field of neuropathy, and there are few studies related to HNSC, which need to be further explored. DNA methyltransferase 1 (methyltransferase1, DNMT1) is a key gene of DNA methylation in mammalian genome epigenetic modification (63), it has the ability to regulate the cell cycle and regulate the expression of tumor suppressor genes, and plays a role in the formation of tumors, Progression, and metastasis. Poor prognosis was all related to the expression level of DNMT1. DNMT1 is highly expressed in a variety of tumors including lung cancer, leukemia, gastric cancer, and liver cancer (64), and the expression of DNMT1 in pancreatic cancer tumor tissue is significantly correlated with the degree of tumor malignancy (65). Thus, these genes play an important role in the development of cancer, which is consistent with the consensus that glucose metabolism plays a role in tumors.

Functional enrichment analyzes revealed the potential biological mechanism of the involved GMRGs. GSEA showed that genes in two groups were mainly enriched in epidermis development, oxidative phosphorylation, and T cell receptor path signaling. T cell receptor signaling pathway plays an important role in T cell mediated immune response, its hyperactivation can lead to autoimmune diseases. On the other hand, defects in TCR signaling can lead to immune deficiency, that contribute to tumor escape (66). As one of the cancer signals, oxidative phosphorylation supports the development of a variety of cancers (67, 68).

Abnormal metabolism is inextricably linked to a dysfunctional immune system in cancer cells (69, 70). Accumulating evidence suggests that immune cell dysfunction in the HNSC microenvironment promotes immunosuppression, correlates with tumor survival and progression, tumor-infiltrating immune cells are dependent on glucose, Impaired immune cell metabolism in the tumor microenvironment contributes to tumor immunological evasion (71, 72). This study showed that there were significant differences in B cells memory, Macrophages M0, Macrophages M2 and Dendritic cells resting in high and low risk groups. Compared with the low-risk group, the infiltration rate of Dendritic resting cells was lower and the infiltration rate of Macrophages M2 was higher in the high-risk group. Dendritic cells are the most critical professional antigen-presenting cells. In the tumor microenvironment, the function and activity of DCs are changed to induce the expansion of regulatory T cells, and the maturation of DCs depends on glycolysis, and the glucose competition of tumor cells will inhibit the activation of DCs (73). Increased infiltration of activated DCs in various tumors is associated with prolonged survival (74–76). Previous studies believed that B cells could promote the occurrence of tumors by regulating the immune response (77). However, the role of memory B cell infiltration in tumor immune response is still unclear. Some studies have found that memory B cells in HNSC patients are significantly reduced. Memory B cells play an important role in tumor memory immune response, which may be due to tumor suppression of its immune environment, sex regulation, and promotion of tumor immune escape (78, 79). Monocytes can bind to a variety of chemokines and be recruited to tumor or inflammatory sites (80). Monocytes infiltrate tumors and differentiate into tumor-associated macrophages (81), tumor-associated dendritic cells, etc (82). It affects the tumor microenvironment through multiple mechanisms, inducing immune tolerance, angiogenesis, and tumor cell metastasis. Tumor-associated macrophages (tumor-associated macrophages, TAM), mainly manifested as M2 type to promote tumor progression through strong immunosuppression (83, 84). The high infiltration of M2 cells is associated with breast cancer, gastric cancer, and hodgkin lymph, and it is related to the poor survival of tumors such as tumors (85–87). According to the subtype of microenvironment, HNSC is divided into two types: active immune response and exhausted immune response. The exhausted immune response type is characterized by high M2 macrophage infiltration, activation of WNT/TGF-β pathway, and poor prognosis. Rich M1 macrophage enrichment, and rich tumor infiltrating lymphocytes are associated with sensitivity to immunotherapy, and usually have a better prognosis (88). Another study divided HNSC into three immune subtypes: ICA, ICB, and ICC. The ICA type is marked by the infiltration of high M1 macrophages, memory CD4 T cells, and CD8 T cells. The immune subtype has a better prognosis. ICB type cluster patients are characterized by markedly increased dendritic cell (DC), activated natural killer (NK) and follicular helper T cell densities and have an intermediate prognosis. Overall survival is shorter in patients with the ICC type, which is characterized by infiltration of stromal components and increased infiltration of M2 macrophage with immunosuppression, and decreased DC infiltration (89). This type is similar to the immune failure type in the previous study, consistent with the conclusions of this study. The results of immune infiltration analysis in this study also showed that TPM2 was positively correlated with macrophage M0 and M2, MTHFD2 was significantly negatively correlated with dendritic resting cells, and DNMT1 was significantly negatively correlated with macrophage M2 and dendritic resting cells. In this study, we found that GMRGs promoted the development of HNSC to some extent. Based on these five key predictors, this study constructed a pre-risk model, divided HNSC patients into high- and low-risk groups after scoring, and found that high-risk groups is associated with poorer prognosis of patients, and its prognostic value is verified, suggesting that the risk score feature can be used as an independent factor to predict the prognosis of patients. In addition, TMB plays an important role in tumors as an indicator of tumor mutational load, reflecting the degree of mutation in the genome of tumor cells, which is closely related to tumor response to immunotherapy and prognosis. But unfortunately, our results showed no difference in TMB between high and low risk groups.

Our study shows that GMRGs (MTHFD2, CDKN2A, TPM2, MPZ and DNMT1) may be a valuable biomarker in the diagnosis of HNSC. We also demonstrate the potential association of MTHFD2, CDKN2A, TPM2, MPZ and DNMT1 and infiltrating immune cells, its important role in the development of HNSC, thereby providing a new insight into the prevention and treatment of HNSC. Despite the demonstrated utility of our model in prognosticating HNSC patients and aiding treatment decisions, our investigation has certain limitations. Primarily, our analysis hinges on data sourced from public databases, which could introduce disparities between predicted outcomes and real-world scenarios. As a result, validation of our model’s clinical effectiveness necessitates the acquisition of prospective clinical information and post-immunotherapy sequencing data. Additionally, the uniqueness of each HNSC patient may influence the characteristics of the GMRGs. To surmount these constraints and bolster the resilience of our model, novel approaches and continued research endeavors are imperative.

## Data availability statement

The original contributions presented in the study are included in the article/**Supplementary Material**. Further inquiries can be directed to the corresponding author.

## Ethics statement

The studies involving humans were approved by the Ethics Committee of Chongqing General Hospital (Ethics No. KYS2021-053-01). The studies were conducted in accordance with the local legislation and institutional requirements. The participants provided their written informed consent to participate in this study.

## Author contributions

YL and ZL conceived the study. YL, NL and XZ drafted the manuscript. LZ and WW performed the literature search and collected the data. YL and JH analyzed and visualized the data. YL and NL completed *in vitro* experiments. ZL helped with the final revision of this manuscript. All authors contributed to the article and approved the submitted version.
