# Supplementary material for: Constructing a prognostic model for head and neck squamous cell carcinoma based on glucose metabolism related genes
Source: Front Endocrinol (Lausanne). 2023 Oct 9;14:1245629. doi: 10.3389/fendo.2023.1245629 (PMC10591078; doi:10.3389/fendo.2023.1245629)
Supplement: Supplementary file 1 [file DataSheet_1.docx]

**Supplementary Table 1. output of vennGen**

| x | x | x | x | x | x | x |
| --- | --- | --- | --- | --- | --- | --- |
| ADH1B | CKS2 | TOP2A | SLC19A1 | FADS1 | TFRC | GSK3B |
| PLIN1 | FBP2 | MBOAT7 | FABP7 | GGH | EGF | GAMT |
| PLIN5 | IGF2BP2 | PLP1 | STMN1 | SLC1A5 | TXNDC9 | LEPR |
| TK1 | SLC2A1 | ACACB | DNMT1 | SHMT2 | DUSP12 | LPL |
| CCNB2 | PPARGC1A | PTTG1 | MTHFD2 | MSH6 | SHMT1 | CXCL8 |
| MAPT | PPARG | CDC25C | RBP1 | UCP3 | GSS | RYR1 |
| SLC2A4 | CA9 | ATIC | BRCA1 | TOMM40 | MAOB | CDKN2A |
| FNDC5 | DNMT3B | AMPD1 | GLA | PPAT | HMGA1 | SLC6A8 |
| MTHFD1L | GPX3 | CTPS1 | SQLE | MPZ | NOS1 | TP73 |
| TUBB | RRM2 | PRKAA2 | TYMS | ACTN3 | FABP3 | GSK3B |
| PDK4 | RBP4 | TPM2 | MEF2C | CD36 | PHGDH |  |

**Supplementary Table 2 Gene and immuneCell correlation**

| gene | immune_cells | cor | p.value |
| --- | --- | --- | --- |
| MTHFD2 | B cells memory | -0.0965 | 0.072594 |
| MTHFD2 | Monocytes | 0.0559 | 0.299102 |
| MTHFD2 | Macrophages M0 | -0.0077 | 0.886351 |
| MTHFD2 | Macrophages M2 | -0.0125 | 0.816525 |
| MTHFD2 | Dendritic cells resting | -0.32305 | 7.18E-10 |
| CDKN2A | B cells memory | 0.050006 | 0.353028 |
| CDKN2A | Monocytes | -0.02476 | 0.645792 |
| CDKN2A | Macrophages M0 | -0.09752 | 0.069608 |
| CDKN2A | Macrophages M2 | -0.10662 | 0.047185 |
| CDKN2A | Dendritic cells resting | -0.07411 | 0.168403 |
| TPM2 | B cells memory | -0.08811 | 0.101283 |
| TPM2 | Monocytes | 0.057996 | 0.281321 |
| TPM2 | Macrophages M0 | 0.168198 | 0.001665 |
| TPM2 | Macrophages M2 | 0.360049 | 4.64E-12 |
| TPM2 | Dendritic cells resting | 0.014969 | 0.781132 |
| MPZ | B cells memory | 0.002625 | 0.961148 |
| MPZ | Monocytes | 0.019313 | 0.719969 |
| MPZ | Macrophages M0 | -0.12527 | 0.019576 |
| MPZ | Macrophages M2 | 0.0173 | 0.74812 |
| MPZ | Dendritic cells resting | -0.06572 | 0.222039 |
| DNMT1 | B cells memory | -0.05747 | 0.285688 |
| DNMT1 | Monocytes | 0.064757 | 0.228899 |
| DNMT1 | Macrophages M0 | -0.13501 | 0.01182 |
| DNMT1 | Macrophages M2 | -0.15355 | 0.004143 |
| DNMT1 | Dendritic cells resting | -0.25003 | 2.41E-06 |

**Supplementary Table 3 Correlation between high and low risk groups and scores.**

|  | risk | StromalScore | ImmuneScore | ESTIMATEScore | TumorPurity |
| --- | --- | --- | --- | --- | --- |
| TCGA-4P-AA8J-01A | High_risk | 578.5839 | 1328.572 | 1907.156 | 0.633339 |
| TCGA-BA-4074-01A | High_risk | 247.9845 | 716.3807 | 964.3652 | 0.73404 |
| TCGA-BA-4075-01A | High_risk | -532.538 | 806.4876 | 273.9496 | 0.79898 |
| TCGA-BA-6871-01A | High_risk | 750.7607 | 1122.3 | 1873.061 | 0.637204 |
| TCGA-BA-A4II-01A | High_risk | -360.168 | 741.2976 | 381.1293 | 0.78942 |
| TCGA-BA-A6D8-01A | High_risk | -225.964 | 525.424 | 299.4602 | 0.796722 |
| TCGA-BA-A6DG-01A | High_risk | 1085.834 | 1310.008 | 2395.842 | 0.576246 |
| TCGA-BA-A8YP-01A | High_risk | 233.6391 | 104.3744 | 338.0135 | 0.793289 |
| TCGA-BB-4217-01A | High_risk | -379.026 | 2259.193 | 1880.167 | 0.6364 |
| TCGA-BB-A5HY-01A | High_risk | -1078.76 | 27.79203 | -1050.97 | 0.900135 |
| TCGA-BB-A5HZ-01A | High_risk | -487.181 | -121.715 | -608.896 | 0.869993 |
| TCGA-BB-A6UO-01A | High_risk | 95.43815 | 616.6849 | 712.1231 | 0.758676 |
| TCGA-CN-4728-01A | High_risk | 1702.228 | 2010.269 | 3712.497 | 0.408543 |
| TCGA-CN-4731-01A | High_risk | -336.48 | 487.0605 | 150.5806 | 0.809738 |
| TCGA-CN-4736-01A | High_risk | 755.2803 | 1964.207 | 2719.488 | 0.536783 |
| TCGA-CN-4737-01A | High_risk | 322.2846 | 477.7973 | 800.0819 | 0.750201 |
| TCGA-CN-4742-01A | High_risk | 615.6433 | 865.9044 | 1481.548 | 0.68042 |
| TCGA-CN-5356-01A | High_risk | 101.9836 | 430.5412 | 532.5248 | 0.775585 |
| TCGA-CN-5358-01A | High_risk | 1766.817 | 2585.79 | 4352.606 | 0.321106 |
| TCGA-CN-5363-01A | High_risk | 325.8656 | 1216.045 | 1541.91 | 0.6739 |
| TCGA-CN-5370-01A | High_risk | 1287.928 | 2004.041 | 3291.969 | 0.464072 |
| TCGA-CN-6017-01A | High_risk | 1240.769 | 2702.777 | 3943.545 | 0.377359 |
| TCGA-CN-6020-01A | High_risk | 336.7519 | 998.9947 | 1335.747 | 0.695946 |
| TCGA-CN-6992-01A | High_risk | -322.358 | 140.3766 | -181.981 | 0.837407 |
| TCGA-CN-A497-01A | High_risk | -395.861 | 608.8933 | 213.0325 | 0.804325 |
| TCGA-CN-A63T-01A | High_risk | -228.536 | 1349.515 | 1120.98 | 0.718235 |
| TCGA-CN-A63U-01A | High_risk | -1618.08 | -397.327 | -2015.41 | 0.952593 |
| TCGA-CN-A63W-01A | High_risk | -1096.58 | 176.182 | -920.398 | 0.891621 |
| TCGA-CN-A642-01A | High_risk | -394.005 | 1205.391 | 811.386 | 0.749103 |
| TCGA-CQ-5325-01A | High_risk | 848.3264 | 2502.326 | 3350.652 | 0.456424 |
| TCGA-CQ-5326-01A | High_risk | -126.248 | 1069.152 | 942.9045 | 0.736175 |
| TCGA-CQ-5332-01A | High_risk | 159.0091 | 134.8566 | 293.8657 | 0.797218 |
| TCGA-CQ-5333-01A | High_risk | 152.3636 | 2408.707 | 2561.071 | 0.556256 |
| TCGA-CQ-6218-01A | High_risk | -66.135 | 975.0167 | 908.8817 | 0.739546 |
| TCGA-CQ-6222-01A | High_risk | 68.43883 | 2056.246 | 2124.685 | 0.60831 |
| TCGA-CQ-6224-01A | High_risk | 1099.135 | 1327.978 | 2427.113 | 0.572488 |
| TCGA-CQ-6227-01A | High_risk | 515.9173 | 643.2567 | 1159.174 | 0.714323 |
| TCGA-CR-5247-01A | High_risk | 275.9457 | -432.231 | -156.285 | 0.835339 |
| TCGA-CR-6478-01A | High_risk | 334.4152 | 2397.203 | 2731.618 | 0.53528 |
| TCGA-CR-7382-01A | High_risk | 1545.641 | 2358.459 | 3904.099 | 0.382714 |
| TCGA-CV-5435-01A | High_risk | -686.649 | -358.956 | -1045.61 | 0.899792 |
| TCGA-CV-5966-01A | High_risk | -168.062 | 1508.781 | 1340.719 | 0.695422 |
| TCGA-CV-5971-01A | High_risk | 1338.649 | 1824.558 | 3163.207 | 0.48073 |
| TCGA-CV-6934-01A | High_risk | 1675.712 | 1759.227 | 3434.939 | 0.445381 |
| TCGA-CV-6937-01A | High_risk | -385.729 | 334.234 | -51.4952 | 0.826785 |
| TCGA-CV-7097-01A | High_risk | 481.7324 | 1252.552 | 1734.285 | 0.652771 |
| TCGA-CV-7102-01A | High_risk | 600.171 | 1007.35 | 1607.521 | 0.666753 |
| TCGA-CV-7177-01A | High_risk | 602.3121 | 144.1264 | 746.4385 | 0.755384 |
| TCGA-CV-7235-01A | High_risk | 822.3093 | 1450.133 | 2272.442 | 0.590954 |
| TCGA-CV-7236-01A | High_risk | 98.74189 | 568.8127 | 667.5546 | 0.762921 |
| TCGA-CV-7252-01A | High_risk | -34.4076 | 856.7309 | 822.3233 | 0.748039 |
| TCGA-CV-7254-01A | High_risk | 1179.653 | 2057.76 | 3237.413 | 0.47115 |
| TCGA-CV-7263-01A | High_risk | 408.4479 | 1110.564 | 1519.012 | 0.676379 |
| TCGA-CV-7421-01A | High_risk | 265.3047 | 222.5962 | 487.9008 | 0.779703 |
| TCGA-CV-7422-01A | High_risk | -140.826 | 684.6654 | 543.839 | 0.774535 |
| TCGA-CV-7424-01A | High_risk | 495.8639 | 788.7805 | 1284.644 | 0.701313 |
| TCGA-CV-7429-01A | High_risk | -30.1239 | 819.4045 | 789.2805 | 0.751249 |
| TCGA-CV-7438-01A | High_risk | -80.5308 | 985.9273 | 905.3965 | 0.73989 |
| TCGA-CV-7446-01A | High_risk | -573.555 | 75.83368 | -497.722 | 0.861831 |
| TCGA-CV-A45Y-01A | High_risk | 618.083 | 1784.414 | 2402.497 | 0.575447 |
| TCGA-CX-7082-01A | High_risk | -511.744 | 242.228 | -269.516 | 0.844361 |
| TCGA-CX-7085-01A | High_risk | 1450.182 | 2466.273 | 3916.455 | 0.381038 |
| TCGA-CX-7219-01A | High_risk | 510.138 | 1318.967 | 1829.105 | 0.642164 |
| TCGA-D6-6826-01A | High_risk | 605.9328 | 1704.091 | 2310.024 | 0.586495 |
| TCGA-D6-6827-01A | High_risk | -686.623 | 85.09685 | -601.526 | 0.869459 |
| TCGA-D6-A6ES-01A | High_risk | -937.946 | 914.9495 | -22.996 | 0.824425 |
| TCGA-DQ-5625-01A | High_risk | -10.8878 | 1859.644 | 1848.757 | 0.63995 |
| TCGA-DQ-5630-01A | High_risk | 737.1642 | 1892.015 | 2629.18 | 0.54792 |
| TCGA-F7-8298-01A | High_risk | -251.023 | 478.1812 | 227.1579 | 0.803091 |
| TCGA-F7-A50I-01A | High_risk | -578.745 | 767.1073 | 188.3626 | 0.806471 |
| TCGA-F7-A61W-01A | High_risk | -1387.48 | -252.673 | -1640.15 | 0.934398 |
| TCGA-F7-A620-01A | High_risk | -382.811 | 1725.486 | 1342.675 | 0.695216 |
| TCGA-F7-A623-01A | High_risk | -926.607 | 484.8856 | -441.721 | 0.857633 |
| TCGA-HD-8635-01A | High_risk | -371.173 | 668.898 | 297.7245 | 0.796876 |
| TCGA-HD-A634-01A | High_risk | -1896.84 | -1052.27 | -2949.11 | 0.985229 |
| TCGA-HD-A6HZ-01A | High_risk | 287.5483 | 1631.503 | 1919.051 | 0.631987 |
| TCGA-HD-A6I0-01A | High_risk | -439.884 | 1244.165 | 804.2812 | 0.749794 |
| TCGA-IQ-A61H-01A | High_risk | -255.969 | 144.5296 | -111.439 | 0.831703 |
| TCGA-IQ-A61O-01A | High_risk | 1482.711 | 1084.309 | 2567.02 | 0.55553 |
| TCGA-KU-A66S-01A | High_risk | -886.826 | -1.35073 | -888.176 | 0.88947 |
| TCGA-KU-A6H8-01A | High_risk | -60.7274 | 789.5914 | 728.8639 | 0.757072 |
| TCGA-MT-A7BN-01A | High_risk | 173.9868 | 1615.018 | 1789.005 | 0.646665 |
| TCGA-MZ-A7D7-01A | High_risk | -1440.75 | -239.842 | -1680.59 | 0.936496 |
| TCGA-QK-A652-01A | High_risk | -214.49 | 1202.61 | 988.1196 | 0.731667 |
| TCGA-QK-A8ZA-01A | High_risk | -1606.07 | -396.974 | -2003.04 | 0.952039 |
| TCGA-QK-AA3K-01A | High_risk | 872.607 | 1270.824 | 2143.431 | 0.606124 |
| TCGA-RS-A6TO-01A | High_risk | -182.228 | 1061.867 | 879.6399 | 0.742429 |
| TCGA-T2-A6WX-01A | High_risk | -618.967 | 2136.078 | 1517.112 | 0.676585 |
| TCGA-T3-A92N-01A | High_risk | -772.953 | 750.6288 | -22.3242 | 0.824369 |
| TCGA-UF-A719-01A | High_risk | -1354.65 | 193.2195 | -1161.43 | 0.90708 |
| TCGA-UF-A71A-01A | High_risk | -55.3785 | -132.868 | -188.246 | 0.837909 |
| TCGA-UF-A71E-01A | High_risk | -984.776 | -390.965 | -1375.74 | 0.919871 |
| TCGA-UF-A7JF-01A | High_risk | -1058.14 | 920.5236 | -137.614 | 0.83383 |
| TCGA-UF-A7JH-01A | High_risk | 497.0145 | 1169.598 | 1666.612 | 0.660264 |
| TCGA-UF-A7JS-01A | High_risk | -370.94 | 1611.978 | 1241.038 | 0.705862 |
| TCGA-UF-A7JV-01A | High_risk | -137.642 | 1529.183 | 1391.542 | 0.690042 |
| TCGA-BA-4078-01A | Low_risk | 406.1951 | 620.0346 | 1026.23 | 0.727843 |
| TCGA-BA-5151-01A | Low_risk | 35.49649 | 1098.717 | 1134.214 | 0.716882 |
| TCGA-BA-5153-01A | Low_risk | -610.405 | 1642.76 | 1032.356 | 0.727226 |
| TCGA-BA-5555-01A | Low_risk | -647.776 | 575.9805 | -71.7952 | 0.828457 |
| TCGA-BA-5556-01A | Low_risk | -152.096 | 2101.743 | 1949.647 | 0.6285 |
| TCGA-BA-5557-01A | Low_risk | 1023.615 | 2217.7 | 3241.315 | 0.470645 |
| TCGA-BA-6868-01B | Low_risk | -1061.03 | -97.1672 | -1158.2 | 0.90688 |
| TCGA-BA-6869-01A | Low_risk | -1324.77 | 368.4029 | -956.37 | 0.893999 |
| TCGA-BA-6870-01A | Low_risk | -619.95 | 1111.59 | 491.6403 | 0.779359 |
| TCGA-BA-6872-01A | Low_risk | -233.543 | 529.3205 | 295.7772 | 0.797049 |
| TCGA-BA-6873-01A | Low_risk | -493.376 | 824.8395 | 331.4635 | 0.793874 |
| TCGA-BA-A4IH-01A | Low_risk | -354.021 | 609.6339 | 255.6133 | 0.800595 |
| TCGA-BA-A6DA-01A | Low_risk | -771.797 | 887.0759 | 115.2793 | 0.812768 |
| TCGA-BA-A6DD-01A | Low_risk | -26.5953 | 1415.121 | 1388.526 | 0.690362 |
| TCGA-BB-4223-01A | Low_risk | -711.281 | 1557.09 | 845.8087 | 0.745746 |
| TCGA-BB-4224-01A | Low_risk | -1456.27 | 108.1799 | -1348.09 | 0.918272 |
| TCGA-BB-8601-01A | Low_risk | -1730.15 | -967.927 | -2698.07 | 0.978251 |
| TCGA-BB-A5HU-01A | Low_risk | 191.641 | 548.8659 | 740.5069 | 0.755955 |
| TCGA-BB-A6UM-01A | Low_risk | -1428.46 | 1100.268 | -328.19 | 0.848944 |
| TCGA-C9-A47Z-01A | Low_risk | -1103.01 | 194.676 | -908.333 | 0.890818 |
| TCGA-CN-4722-01A | Low_risk | 333.9504 | 457.8138 | 791.7642 | 0.751008 |
| TCGA-CN-4723-01A | Low_risk | -79.7219 | 958.8596 | 879.1377 | 0.742478 |
| TCGA-CN-4725-01A | Low_risk | -820.737 | -151.482 | -972.219 | 0.895039 |
| TCGA-CN-4729-01A | Low_risk | -141.311 | 645.5808 | 504.2702 | 0.778196 |
| TCGA-CN-4733-01A | Low_risk | 1242.554 | 2790.949 | 4033.503 | 0.365098 |
| TCGA-CN-4734-01A | Low_risk | 139.3444 | 1675.075 | 1814.419 | 0.643815 |
| TCGA-CN-4735-01A | Low_risk | -39.8631 | 1651.424 | 1611.561 | 0.666311 |
| TCGA-CN-4741-01A | Low_risk | -919.511 | 382.9538 | -536.557 | 0.864708 |
| TCGA-CN-5355-01A | Low_risk | 381.1341 | 449.0308 | 830.1649 | 0.747274 |
| TCGA-CN-5359-01A | Low_risk | 1267.812 | 2123.118 | 3390.93 | 0.451156 |
| TCGA-CN-5360-01A | Low_risk | 368.1139 | 1944.234 | 2312.348 | 0.586218 |
| TCGA-CN-5361-01A | Low_risk | 742.3527 | 691.6749 | 1434.028 | 0.685515 |
| TCGA-CN-5366-01A | Low_risk | 186.4661 | 881.5947 | 1068.061 | 0.723618 |
| TCGA-CN-5367-01A | Low_risk | -293.741 | 726.2739 | 432.5331 | 0.784766 |
| TCGA-CN-5373-01A | Low_risk | 628.2278 | 1616.809 | 2245.036 | 0.594195 |
| TCGA-CN-6011-01A | Low_risk | -653.396 | 700.9298 | 47.5338 | 0.818521 |
| TCGA-CN-6016-01A | Low_risk | 1215.724 | 1369.582 | 2585.307 | 0.553296 |
| TCGA-CN-6019-01A | Low_risk | 1627.772 | 1949.967 | 3577.74 | 0.426517 |
| TCGA-CN-6021-01A | Low_risk | -591.886 | 515.9706 | -75.9149 | 0.828796 |
| TCGA-CN-6023-01A | Low_risk | -543.004 | 1524.711 | 981.7073 | 0.732309 |
| TCGA-CN-6024-01A | Low_risk | -434.791 | -620.937 | -1055.73 | 0.900439 |
| TCGA-CN-6988-01A | Low_risk | -1505.36 | -603.962 | -2109.32 | 0.956696 |
| TCGA-CN-6996-01A | Low_risk | 326.2232 | 1888.919 | 2215.142 | 0.597718 |
| TCGA-CN-6997-01A | Low_risk | -509.184 | -131.602 | -640.785 | 0.872291 |
| TCGA-CN-6998-01A | Low_risk | -929.817 | 451.8941 | -477.923 | 0.860353 |
| TCGA-CN-A49B-01A | Low_risk | -221.412 | 1616.722 | 1395.309 | 0.689642 |
| TCGA-CN-A49C-01A | Low_risk | -1550.21 | 487.0835 | -1063.13 | 0.900911 |
| TCGA-CN-A63V-01A | Low_risk | 530.0453 | 453.1469 | 983.1922 | 0.73216 |
| TCGA-CN-A641-01A | Low_risk | -1234.28 | 21.35901 | -1212.92 | 0.910235 |
| TCGA-CN-A6UY-01A | Low_risk | -1138.34 | 1761.573 | 623.2353 | 0.767111 |
| TCGA-CN-A6V7-01A | Low_risk | -2040.53 | 275.6326 | -1764.89 | 0.940764 |
| TCGA-CQ-5329-01A | Low_risk | 406.112 | 1351.014 | 1757.126 | 0.650227 |
| TCGA-CQ-5330-01A | Low_risk | 882.3494 | 1884.255 | 2766.604 | 0.530935 |
| TCGA-CQ-5334-01A | Low_risk | 726.9099 | 1827.191 | 2554.101 | 0.557106 |
| TCGA-CQ-6219-01A | Low_risk | 485.495 | 1766.234 | 2251.729 | 0.593404 |
| TCGA-CQ-6221-01A | Low_risk | 1151.864 | 825.2091 | 1977.073 | 0.625364 |
| TCGA-CQ-6223-01A | Low_risk | -884.603 | 863.8205 | -20.7828 | 0.824241 |
| TCGA-CQ-6225-01A | Low_risk | -2013.16 | -415.124 | -2428.29 | 0.969272 |
| TCGA-CQ-6228-01A | Low_risk | -352.729 | 167.6509 | -185.078 | 0.837655 |
| TCGA-CQ-6229-01A | Low_risk | -290.704 | 797.797 | 507.0934 | 0.777936 |
| TCGA-CQ-7063-01A | Low_risk | -396.601 | 1924.522 | 1527.92 | 0.675416 |
| TCGA-CQ-7065-01A | Low_risk | -1161.12 | -603.432 | -1764.56 | 0.940747 |
| TCGA-CQ-7068-01A | Low_risk | -663.213 | 1903.529 | 1240.316 | 0.705937 |
| TCGA-CQ-7069-01A | Low_risk | -328.638 | 375.496 | 46.85844 | 0.818578 |
| TCGA-CQ-7071-01A | Low_risk | -25.4173 | 1102.75 | 1077.333 | 0.722678 |
| TCGA-CQ-A4C6-01A | Low_risk | -192.544 | 1009.881 | 817.337 | 0.748524 |
| TCGA-CQ-A4C7-01A | Low_risk | 241.2555 | 444.6795 | 685.9351 | 0.761174 |
| TCGA-CQ-A4CB-01A | Low_risk | 446.7017 | 1005.034 | 1451.736 | 0.68362 |
| TCGA-CQ-A4CD-01A | Low_risk | -584.014 | 1140.017 | 556.0038 | 0.773404 |
| TCGA-CQ-A4CE-01A | Low_risk | -890.991 | 730.7328 | -160.259 | 0.83566 |
| TCGA-CQ-A4CI-01A | Low_risk | -378.282 | -591.079 | -969.361 | 0.894852 |
| TCGA-CR-5243-01A | Low_risk | 27.67221 | 1109.821 | 1137.493 | 0.716546 |
| TCGA-CR-5248-01A | Low_risk | -374.983 | 2735.269 | 2360.286 | 0.580503 |
| TCGA-CR-5249-01A | Low_risk | -454.769 | 1962.125 | 1507.356 | 0.677639 |
| TCGA-CR-5250-01A | Low_risk | -442.359 | 1130.905 | 688.5466 | 0.760926 |
| TCGA-CR-6470-01A | Low_risk | 328.7383 | 2224.361 | 2553.1 | 0.557228 |
| TCGA-CR-6471-01A | Low_risk | 243.1445 | 1593.626 | 1836.771 | 0.641301 |
| TCGA-CR-6473-01A | Low_risk | -1107.1 | 125.2126 | -981.89 | 0.895672 |
| TCGA-CR-6474-01A | Low_risk | 1041.251 | 1341.459 | 2382.71 | 0.57782 |
| TCGA-CR-6477-01A | Low_risk | 606.9167 | 1499.588 | 2106.504 | 0.610426 |
| TCGA-CR-6481-01A | Low_risk | 344.2138 | 2875.915 | 3220.129 | 0.473387 |
| TCGA-CR-6482-01A | Low_risk | 353.1428 | 2459.661 | 2812.803 | 0.525176 |
| TCGA-CR-6484-01A | Low_risk | 911.5654 | 2221.303 | 3132.869 | 0.48463 |
| TCGA-CR-6487-01A | Low_risk | 284.6467 | 2740.812 | 3025.459 | 0.498361 |
| TCGA-CR-6492-01A | Low_risk | -520.431 | 951.7951 | 431.3643 | 0.784872 |
| TCGA-CR-7364-01A | Low_risk | -461.553 | 599.6407 | 138.0876 | 0.810813 |
| TCGA-CR-7365-01A | Low_risk | 80.73486 | 281.9717 | 362.7065 | 0.791077 |
| TCGA-CR-7367-01A | Low_risk | 997.9276 | 1071.716 | 2069.644 | 0.614703 |
| TCGA-CR-7368-01A | Low_risk | -592.861 | 969.9495 | 377.0888 | 0.789784 |
| TCGA-CR-7369-01A | Low_risk | -195.501 | 664.2118 | 468.7109 | 0.781463 |
| TCGA-CR-7370-01A | Low_risk | -201.296 | 215.9558 | 14.65994 | 0.821284 |
| TCGA-CR-7373-01A | Low_risk | -363.859 | 1685.024 | 1321.165 | 0.697482 |
| TCGA-CR-7374-01A | Low_risk | -682.924 | 697.1883 | 14.26481 | 0.821317 |
| TCGA-CR-7377-01A | Low_risk | 896.2433 | 2010.632 | 2906.875 | 0.513376 |
| TCGA-CR-7385-01A | Low_risk | -784.004 | 1877.152 | 1093.147 | 0.721072 |
| TCGA-CR-7386-01A | Low_risk | 1511.298 | 1971.197 | 3482.495 | 0.43912 |
| TCGA-CR-7388-01A | Low_risk | 275.3357 | 1642.825 | 1918.16 | 0.632088 |
| TCGA-CR-7389-01A | Low_risk | 385.244 | 953.8298 | 1339.074 | 0.695596 |
| TCGA-CR-7390-01A | Low_risk | -219.396 | 42.43609 | -176.96 | 0.837004 |
| TCGA-CR-7391-01A | Low_risk | 833.8296 | 1939.998 | 2773.827 | 0.530036 |
| TCGA-CR-7393-01A | Low_risk | 1220.074 | 2346.924 | 3566.998 | 0.427943 |
| TCGA-CR-7394-01A | Low_risk | 815.5773 | 2363.187 | 3178.764 | 0.478726 |
| TCGA-CR-7397-01A | Low_risk | 1204.591 | 1088.231 | 2292.822 | 0.588538 |
| TCGA-CR-7401-01A | Low_risk | 388.9009 | 728.428 | 1117.329 | 0.718608 |
| TCGA-CR-7402-01A | Low_risk | -691.586 | 656.0161 | -35.5699 | 0.825468 |
| TCGA-CV-5430-01A | Low_risk | -228.403 | 1239.524 | 1011.122 | 0.729361 |
| TCGA-CV-5431-01A | Low_risk | 577.5998 | 2002.094 | 2579.693 | 0.553982 |
| TCGA-CV-5432-01A | Low_risk | -789.444 | 115.2646 | -674.18 | 0.874678 |
| TCGA-CV-5436-01A | Low_risk | -585.315 | 355.399 | -229.916 | 0.841232 |
| TCGA-CV-5440-01A | Low_risk | -1016.81 | -161.891 | -1178.7 | 0.908144 |
| TCGA-CV-5442-01A | Low_risk | -1881.05 | -414.027 | -2295.08 | 0.964277 |
| TCGA-CV-5443-01A | Low_risk | -85.9575 | 1919.943 | 1833.985 | 0.641615 |
| TCGA-CV-5444-01A | Low_risk | 1235.402 | 1670.07 | 2905.472 | 0.513552 |
| TCGA-CV-5970-01A | Low_risk | 117.8594 | 694.4993 | 812.3587 | 0.749008 |
| TCGA-CV-5973-01A | Low_risk | -1285.68 | 388.3086 | -897.374 | 0.890086 |
| TCGA-CV-5976-01A | Low_risk | -509.647 | 269.811 | -239.836 | 0.842019 |
| TCGA-CV-5977-01A | Low_risk | -988.403 | 175.8672 | -812.536 | 0.884341 |
| TCGA-CV-5979-01A | Low_risk | -895.223 | 300.3528 | -594.87 | 0.868976 |
| TCGA-CV-6003-01A | Low_risk | -1122.8 | 124.7949 | -998.006 | 0.896721 |
| TCGA-CV-6433-01A | Low_risk | -865.12 | 2001.256 | 1136.136 | 0.716685 |
| TCGA-CV-6935-01A | Low_risk | -85.8327 | 40.14484 | -45.6879 | 0.826305 |
| TCGA-CV-6936-01A | Low_risk | -605.923 | 780.4399 | 174.5165 | 0.807671 |
| TCGA-CV-6939-01A | Low_risk | -159.743 | 1346.274 | 1186.531 | 0.711507 |
| TCGA-CV-6941-01A | Low_risk | 342.9239 | 544.3035 | 887.2275 | 0.741682 |
| TCGA-CV-6942-01A | Low_risk | 784.1201 | 2150.504 | 2934.625 | 0.509876 |
| TCGA-CV-6943-01A | Low_risk | 1272.786 | 2544.906 | 3817.691 | 0.394401 |
| TCGA-CV-6945-01A | Low_risk | -928.127 | -98.1154 | -1026.24 | 0.898548 |
| TCGA-CV-6950-01A | Low_risk | -250.903 | 441.9964 | 191.0933 | 0.806234 |
| TCGA-CV-6951-01A | Low_risk | -926.895 | -272.91 | -1199.81 | 0.909436 |
| TCGA-CV-6954-01A | Low_risk | 61.65321 | 818.0457 | 879.6989 | 0.742423 |
| TCGA-CV-6955-01A | Low_risk | -1049.08 | 1428.886 | 379.8066 | 0.789539 |
| TCGA-CV-6959-01A | Low_risk | -64.0079 | 1124.216 | 1060.208 | 0.724413 |
| TCGA-CV-6961-01A | Low_risk | -71.4944 | 590.8474 | 519.3529 | 0.776804 |
| TCGA-CV-7089-01A | Low_risk | -961.979 | 692.5493 | -269.43 | 0.844354 |
| TCGA-CV-7090-01A | Low_risk | 587.4702 | 1607.669 | 2195.139 | 0.60007 |
| TCGA-CV-7091-01A | Low_risk | -68.6092 | 679.984 | 611.3748 | 0.768227 |
| TCGA-CV-7095-01A | Low_risk | -709.316 | 439.241 | -270.075 | 0.844405 |
| TCGA-CV-7103-01A | Low_risk | 784.7877 | 61.34603 | 846.1338 | 0.745714 |
| TCGA-CV-7104-01A | Low_risk | 243.2704 | 1313.833 | 1557.103 | 0.67225 |
| TCGA-CV-7178-01A | Low_risk | 752.1086 | 1396.284 | 2148.392 | 0.605545 |
| TCGA-CV-7180-01A | Low_risk | -1492.13 | 181.5486 | -1310.58 | 0.916078 |
| TCGA-CV-7242-01A | Low_risk | -1037.5 | 1345.046 | 307.5416 | 0.796005 |
| TCGA-CV-7247-01A | Low_risk | -456.262 | -318.297 | -774.559 | 0.881725 |
| TCGA-CV-7250-01A | Low_risk | -1045.47 | 395.8034 | -649.662 | 0.872928 |
| TCGA-CV-7253-01A | Low_risk | -1751.13 | -447.994 | -2199.13 | 0.96045 |
| TCGA-CV-7255-01A | Low_risk | -1337.08 | -314.949 | -1652.03 | 0.935017 |
| TCGA-CV-7407-01A | Low_risk | -782.035 | 1379.265 | 597.2301 | 0.769554 |
| TCGA-CV-7410-01A | Low_risk | 501.7702 | 2717.146 | 3218.917 | 0.473543 |
| TCGA-CV-7411-01A | Low_risk | -38.7519 | 490.7539 | 452.002 | 0.782991 |
| TCGA-CV-7414-01A | Low_risk | -279.411 | 229.9687 | -49.4428 | 0.826615 |
| TCGA-CV-7416-01A | Low_risk | 275.8332 | 510.1531 | 785.9863 | 0.751568 |
| TCGA-CV-7425-01A | Low_risk | -437.012 | 1051.444 | 614.4318 | 0.767939 |
| TCGA-CV-7427-01A | Low_risk | -423.866 | 1830.277 | 1406.41 | 0.688461 |
| TCGA-CV-7428-01A | Low_risk | -230.415 | 1258.971 | 1028.555 | 0.727608 |
| TCGA-CV-7432-01A | Low_risk | -982.847 | 166.2714 | -816.576 | 0.884617 |
| TCGA-CV-7434-01A | Low_risk | -580.458 | 720.7935 | 140.3356 | 0.81062 |
| TCGA-CV-7435-01A | Low_risk | 1156.216 | 1119.928 | 2276.144 | 0.590516 |
| TCGA-CV-7437-01A | Low_risk | -1012.24 | -68.1284 | -1080.37 | 0.902007 |
| TCGA-CV-7568-01A | Low_risk | 211.0402 | 171.5159 | 382.5561 | 0.789292 |
| TCGA-CV-A45O-01A | Low_risk | -648.855 | 1048.048 | 399.193 | 0.78779 |
| TCGA-CV-A45T-01A | Low_risk | -6.36168 | 2355.766 | 2349.404 | 0.581803 |
| TCGA-CV-A45U-01A | Low_risk | -1036.88 | 294.4372 | -742.439 | 0.87949 |
| TCGA-CV-A45V-01A | Low_risk | -43.3911 | 1045.382 | 1001.991 | 0.730278 |
| TCGA-CV-A45X-01A | Low_risk | -1231.24 | 240.0299 | -991.209 | 0.896279 |
| TCGA-CV-A45Z-01A | Low_risk | -458.818 | 1103.458 | 644.6404 | 0.765091 |
| TCGA-CV-A463-01A | Low_risk | -1108.03 | 265.9778 | -842.052 | 0.886355 |
| TCGA-CV-A465-01A | Low_risk | -1498.99 | -346.301 | -1845.29 | 0.944699 |
| TCGA-CV-A468-01A | Low_risk | 745.693 | 2091.475 | 2837.167 | 0.522129 |
| TCGA-CV-A6JD-01A | Low_risk | -87.5494 | 1731.721 | 1644.171 | 0.662734 |
| TCGA-CV-A6JE-01A | Low_risk | -16.1483 | 1417.888 | 1401.74 | 0.688958 |
| TCGA-CV-A6JM-01A | Low_risk | -670.851 | 659.0641 | -11.7866 | 0.823492 |
| TCGA-CV-A6JN-01A | Low_risk | -808.749 | 1195.062 | 386.3129 | 0.788953 |
| TCGA-CV-A6JO-01B | Low_risk | -557.521 | 1455.431 | 897.9098 | 0.740629 |
| TCGA-CV-A6JT-01A | Low_risk | 171.2091 | 1191.751 | 1362.96 | 0.693072 |
| TCGA-CV-A6JY-01A | Low_risk | -1248.11 | -83.2564 | -1331.36 | 0.917297 |
| TCGA-CV-A6JZ-01A | Low_risk | 423.6624 | 2144.012 | 2567.675 | 0.55545 |
| TCGA-CV-A6K0-01B | Low_risk | 534.8694 | 1870.525 | 2405.394 | 0.575099 |
| TCGA-CV-A6K1-01A | Low_risk | -597.89 | 882.1478 | 284.2576 | 0.798069 |
| TCGA-CX-7086-01A | Low_risk | -1251.35 | 250.3371 | -1001.01 | 0.896917 |
| TCGA-CX-A4AQ-01A | Low_risk | -248.541 | 2064.35 | 1815.809 | 0.643659 |
| TCGA-D6-6515-01A | Low_risk | 1098.734 | 2357.496 | 3456.23 | 0.442581 |
| TCGA-D6-6517-01A | Low_risk | -542.832 | 952.6605 | 409.8286 | 0.786827 |
| TCGA-D6-6824-01A | Low_risk | 784.0603 | 1789.909 | 2573.969 | 0.554682 |
| TCGA-D6-6825-01A | Low_risk | 364.0782 | 1992.108 | 2356.186 | 0.580993 |
| TCGA-D6-8568-01A | Low_risk | 152.8708 | 994.3066 | 1147.177 | 0.715554 |
| TCGA-D6-8569-01A | Low_risk | -415.551 | 780.6256 | 365.0746 | 0.790865 |
| TCGA-D6-A4ZB-01A | Low_risk | -732.647 | 444.9123 | -287.735 | 0.845791 |
| TCGA-D6-A6EK-01A | Low_risk | -499.791 | 1806.764 | 1306.973 | 0.698973 |
| TCGA-D6-A6EM-01A | Low_risk | -275.835 | 1921.624 | 1645.789 | 0.662556 |
| TCGA-D6-A6EN-01A | Low_risk | -643.416 | 2187.48 | 1544.064 | 0.673666 |
| TCGA-D6-A6EO-01A | Low_risk | -976.329 | -151.93 | -1128.26 | 0.905019 |
| TCGA-D6-A6EQ-01A | Low_risk | 44.27562 | 1381.584 | 1425.859 | 0.686387 |
| TCGA-D6-A74Q-01A | Low_risk | -696.464 | 781.0608 | 84.59694 | 0.815383 |
| TCGA-DQ-5629-01A | Low_risk | -691.543 | -384.678 | -1076.22 | 0.901743 |
| TCGA-DQ-5631-01A | Low_risk | -547.721 | -12.7557 | -560.477 | 0.866467 |
| TCGA-DQ-7591-01A | Low_risk | -1145.7 | 1737.161 | 591.4605 | 0.770095 |
| TCGA-DQ-7592-01A | Low_risk | -143.511 | 782.689 | 639.1776 | 0.765608 |
| TCGA-F7-7848-01A | Low_risk | -106.688 | 1151.619 | 1044.931 | 0.725957 |
| TCGA-F7-A50G-01A | Low_risk | -324.011 | 1424.472 | 1100.461 | 0.720327 |
| TCGA-F7-A61S-01A | Low_risk | -911.475 | 1404.56 | 493.0851 | 0.779226 |
| TCGA-F7-A61V-01A | Low_risk | -1129.31 | 179.5524 | -949.754 | 0.893564 |
| TCGA-H7-7774-01A | Low_risk | -983.589 | 957.6375 | -25.9516 | 0.82467 |
| TCGA-H7-A76A-01A | Low_risk | 700.0677 | 2764.46 | 3464.528 | 0.441488 |
| TCGA-HD-7229-01A | Low_risk | -1051.43 | 995.7657 | -55.6665 | 0.827129 |
| TCGA-HD-7753-01A | Low_risk | -78.6037 | 721.1787 | 642.5749 | 0.765287 |
| TCGA-HD-7754-01A | Low_risk | 307.7396 | 389.4485 | 697.1881 | 0.760102 |
| TCGA-HD-7831-01A | Low_risk | 1267.95 | 596.5383 | 1864.489 | 0.638174 |
| TCGA-HD-7832-01A | Low_risk | -670.46 | 799.9556 | 129.4955 | 0.81155 |
| TCGA-HD-8634-01A | Low_risk | -987.316 | 442.0799 | -545.237 | 0.865348 |
| TCGA-HD-A4C1-01A | Low_risk | -499.852 | 273.0985 | -226.754 | 0.840981 |
| TCGA-HD-A633-01A | Low_risk | -756.392 | 241.8467 | -514.546 | 0.863081 |
| TCGA-HL-7533-01A | Low_risk | -850.055 | 1154.923 | 304.8688 | 0.796242 |
| TCGA-IQ-7630-01A | Low_risk | -429.423 | 816.1443 | 386.7214 | 0.788916 |
| TCGA-IQ-7631-01A | Low_risk | 543.8549 | -151.2 | 392.6546 | 0.78838 |
| TCGA-IQ-7632-01A | Low_risk | -1214.83 | 73.63236 | -1141.2 | 0.905825 |
| TCGA-IQ-A61I-01A | Low_risk | -1241.27 | 1336.713 | 95.44066 | 0.814461 |
| TCGA-IQ-A61J-01A | Low_risk | 203.5207 | 1054.403 | 1257.924 | 0.704104 |
| TCGA-IQ-A6SG-01A | Low_risk | -1066.18 | -171.202 | -1237.38 | 0.911716 |
| TCGA-IQ-A6SH-01A | Low_risk | -459.606 | 1227.178 | 767.5719 | 0.753348 |
| TCGA-KU-A6H7-01A | Low_risk | -790.955 | 1809.614 | 1018.659 | 0.728604 |
| TCGA-MT-A51X-01A | Low_risk | -450.597 | 335.587 | -115.01 | 0.831993 |
| TCGA-MT-A67A-01A | Low_risk | -1046.22 | 630.7698 | -415.448 | 0.855643 |
| TCGA-MT-A67D-01A | Low_risk | 76.44256 | 1167.32 | 1243.762 | 0.705579 |
| TCGA-MT-A67F-01A | Low_risk | 162.0824 | 2684.158 | 2846.24 | 0.520993 |
| TCGA-MZ-A5BI-01A | Low_risk | -337.242 | 2390.292 | 2053.051 | 0.616622 |
| TCGA-P3-A5QE-01A | Low_risk | -1379.86 | 1828.467 | 448.6112 | 0.783301 |
| TCGA-P3-A5QF-01A | Low_risk | -1048.14 | 493.4244 | -554.714 | 0.866044 |
| TCGA-P3-A6SW-01A | Low_risk | -515.628 | 1420.65 | 905.0229 | 0.739927 |
| TCGA-P3-A6T0-01A | Low_risk | -856.082 | -223.521 | -1079.6 | 0.901958 |
| TCGA-P3-A6T3-01A | Low_risk | -960.158 | -115.951 | -1076.11 | 0.901736 |
| TCGA-P3-A6T5-01A | Low_risk | -268.069 | 626.9815 | 358.9123 | 0.791418 |
| TCGA-P3-A6T8-01A | Low_risk | 205.3152 | 0.966895 | 206.2821 | 0.804913 |
| TCGA-QK-A64Z-01A | Low_risk | -617.17 | 422.5495 | -194.621 | 0.83842 |
| TCGA-QK-A6IF-01A | Low_risk | -690.754 | 2231.526 | 1540.772 | 0.674023 |
| TCGA-QK-A6IH-01A | Low_risk | -484.34 | 439.4314 | -44.9086 | 0.826241 |
| TCGA-QK-A6II-01A | Low_risk | -1189.83 | 801.6511 | -388.177 | 0.853564 |
| TCGA-QK-A6IJ-01A | Low_risk | -1001.09 | 1364.61 | 363.5242 | 0.791004 |
| TCGA-QK-A6V9-01A | Low_risk | -1597.06 | 1288.919 | -308.14 | 0.847385 |
| TCGA-QK-A6VC-01A | Low_risk | -247.223 | 1551.89 | 1304.667 | 0.699215 |
| TCGA-QK-A8Z9-01B | Low_risk | 993.0869 | 1085.877 | 2078.964 | 0.613623 |
| TCGA-QK-AA3J-01A | Low_risk | -1590.5 | 260.1461 | -1330.36 | 0.917238 |
| TCGA-RS-A6TP-01A | Low_risk | -1730.37 | 408.2844 | -1322.08 | 0.916753 |
| TCGA-T2-A6WZ-01A | Low_risk | -659.864 | 781.8106 | 121.9469 | 0.812197 |
| TCGA-T2-A6X2-01A | Low_risk | -188.579 | 1106.815 | 918.2365 | 0.738621 |
| TCGA-T3-A92M-01A | Low_risk | 304.5113 | 1245.163 | 1549.675 | 0.673057 |
| TCGA-UF-A718-01A | Low_risk | -52.0107 | 807.0235 | 755.0128 | 0.754559 |
| TCGA-UF-A71D-01A | Low_risk | -1267.97 | -5.78667 | -1273.75 | 0.913896 |
| TCGA-UF-A7J9-01A | Low_risk | 688.3779 | 589.5604 | 1277.938 | 0.702015 |
| TCGA-UF-A7JC-01A | Low_risk | -1164.91 | -295.156 | -1460.07 | 0.924656 |
| TCGA-UF-A7JK-01A | Low_risk | -1121.54 | -610.914 | -1732.45 | 0.939138 |
| TCGA-UF-A7JT-01A | Low_risk | 205.1269 | 2145.224 | 2350.35 | 0.58169 |
| TCGA-UP-A6WW-01A | Low_risk | -350.902 | 1118.796 | 767.8946 | 0.753317 |
| TCGA-WA-A7GZ-01A | Low_risk | -771.655 | -120.84 | -892.495 | 0.889759 |
| TCGA-WA-A7H4-01A | Low_risk | -39.3189 | 481.9597 | 442.6408 | 0.783845 |

**
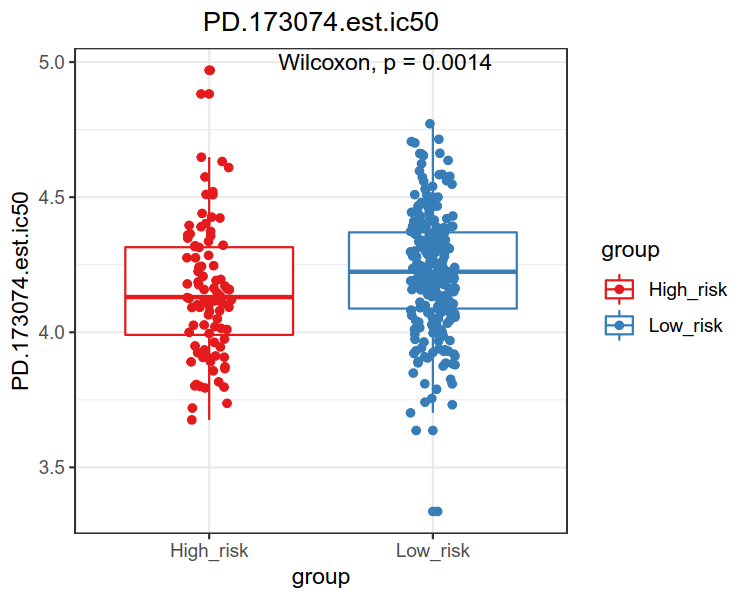

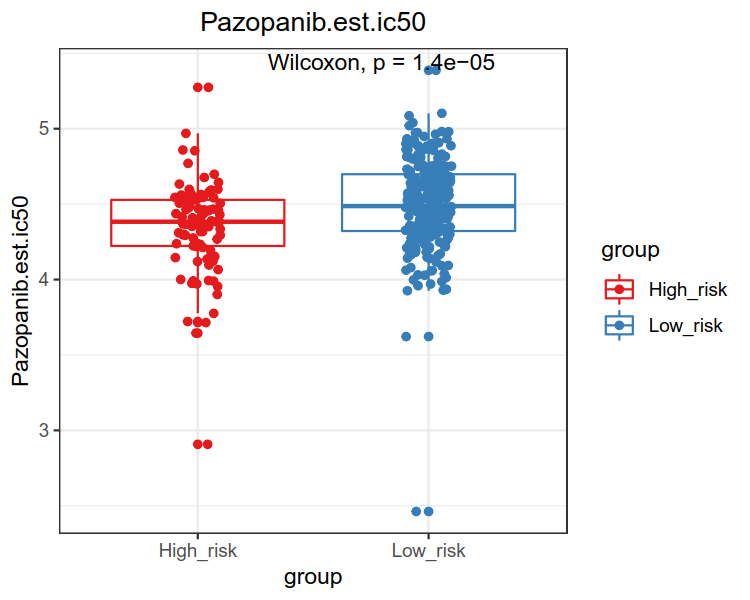

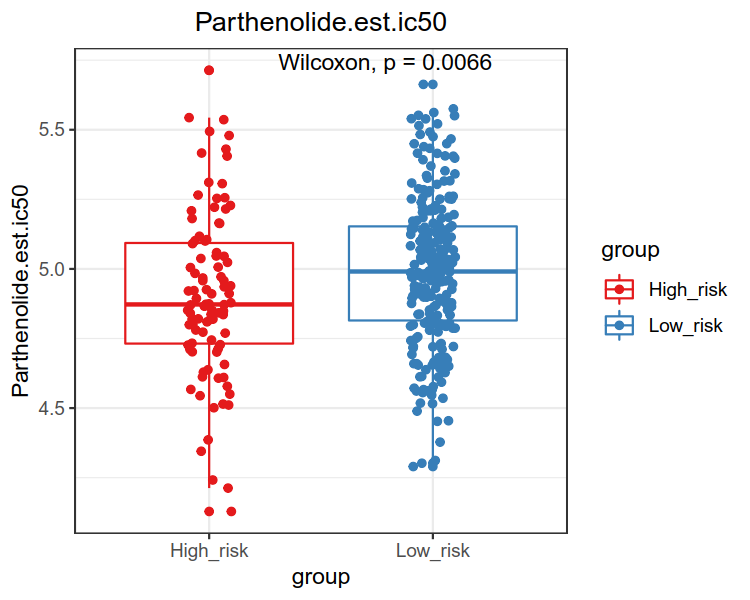

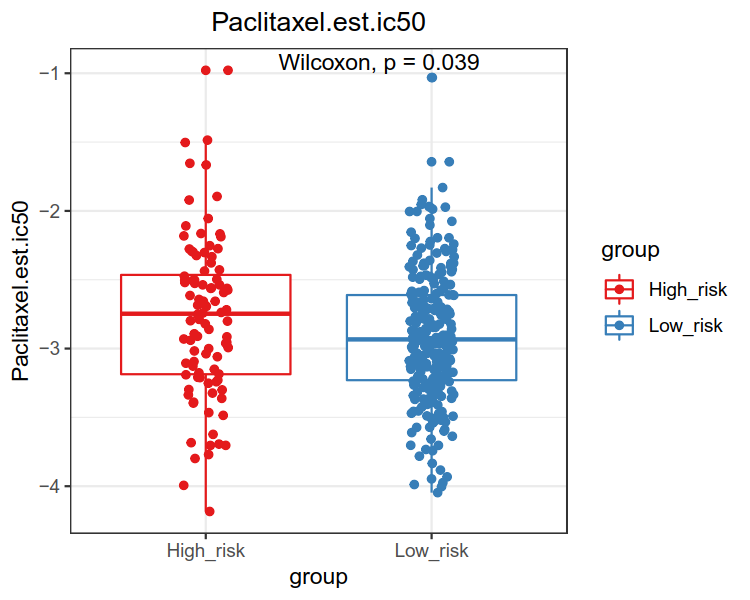

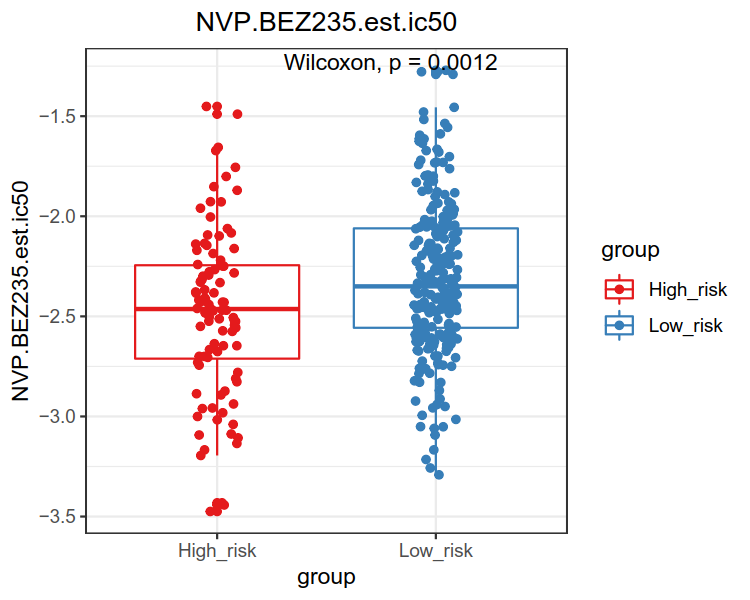

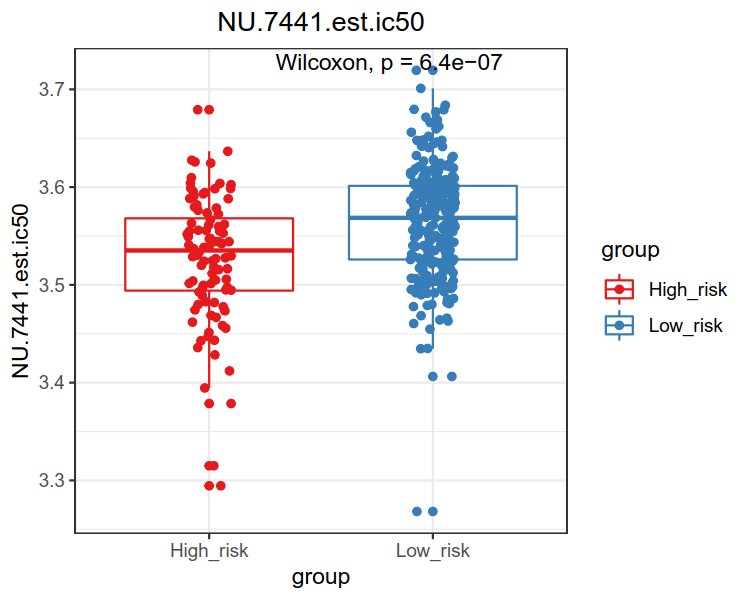

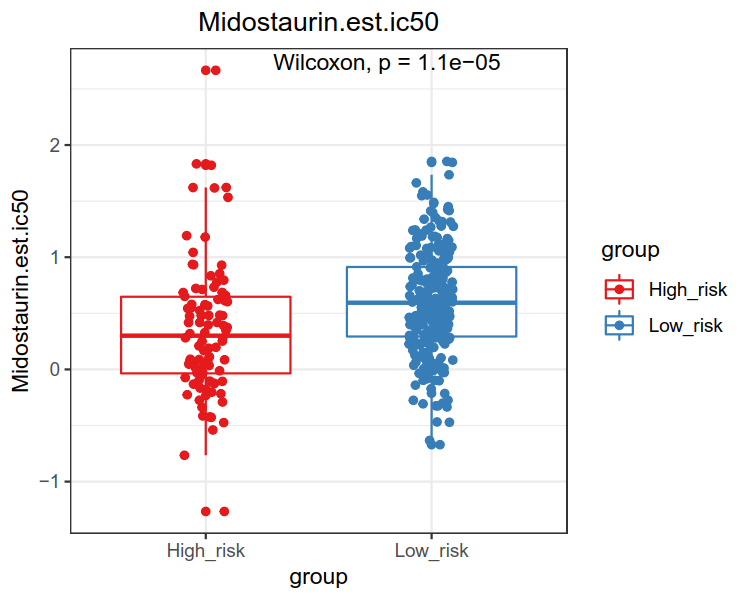

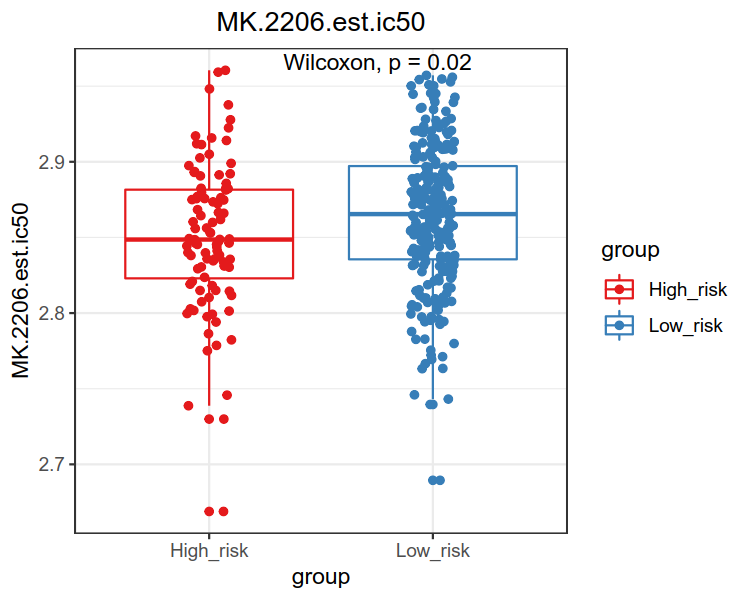

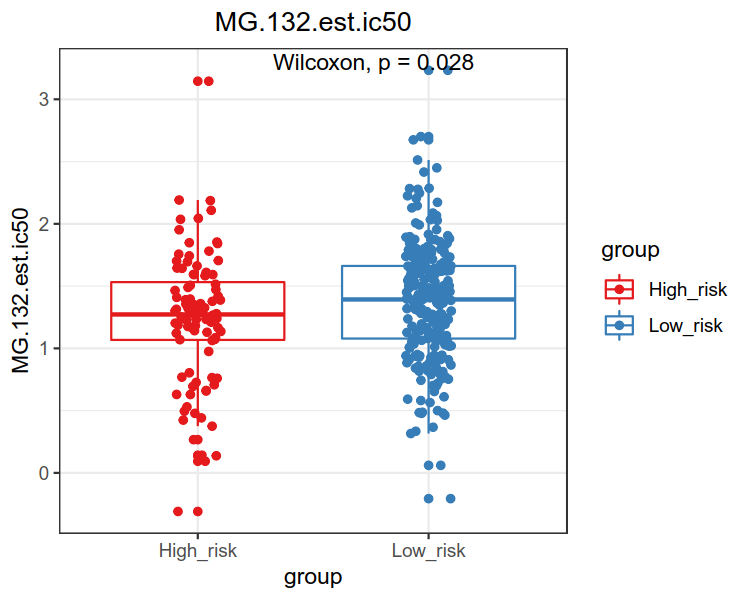

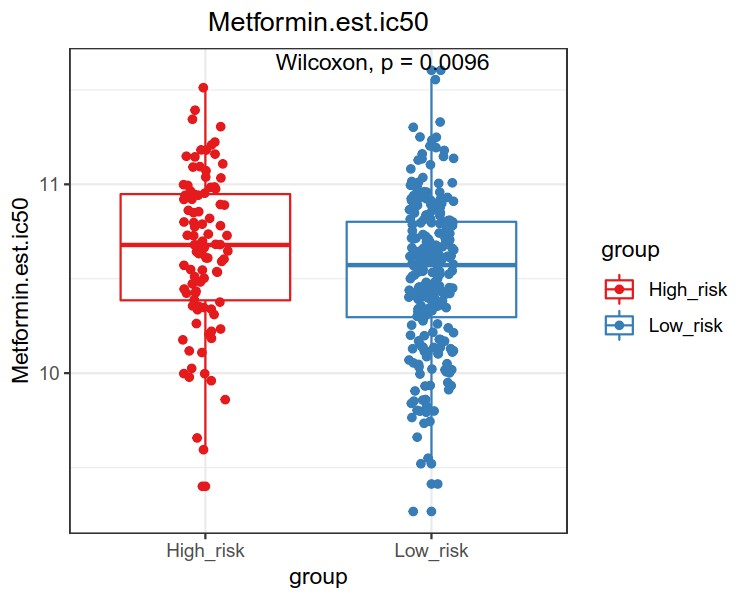

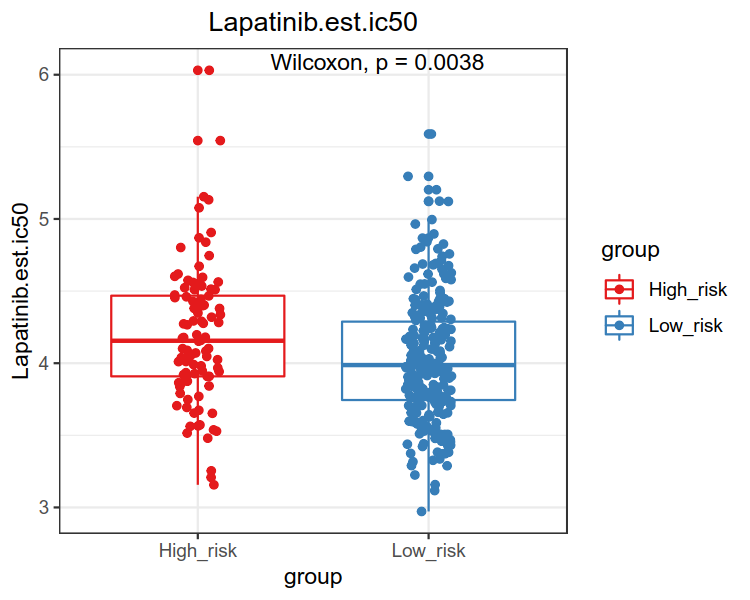

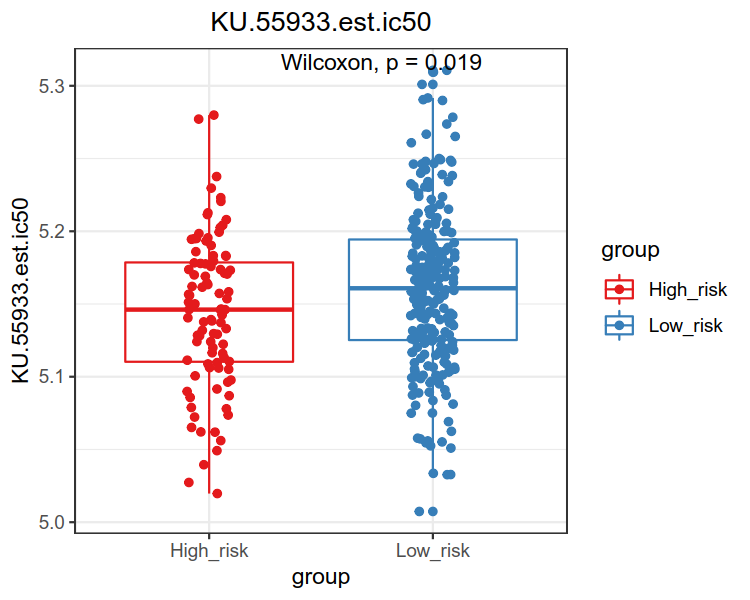

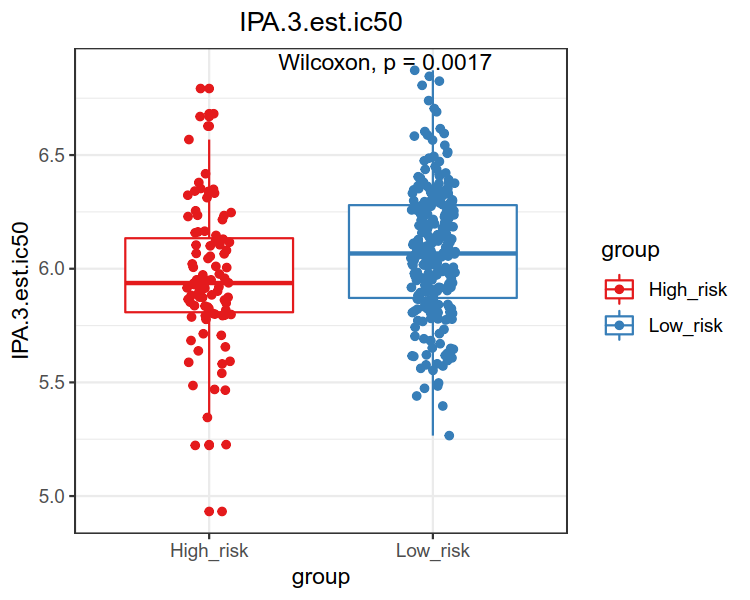

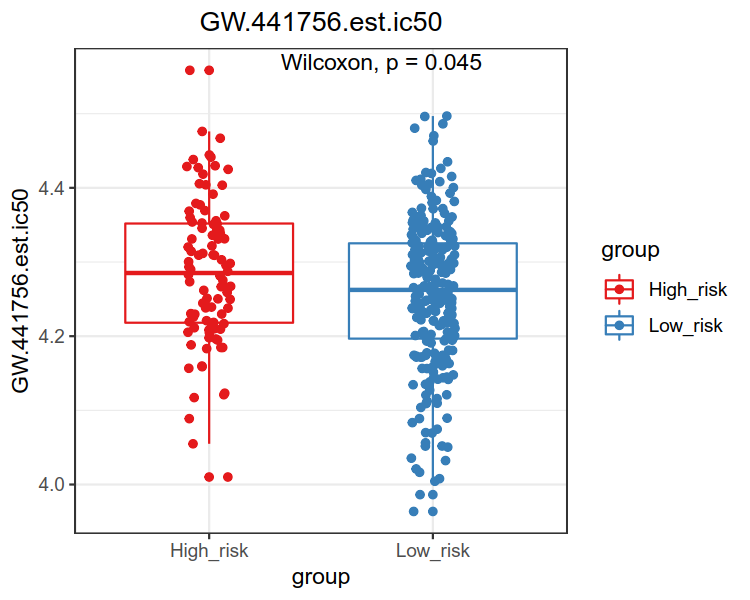

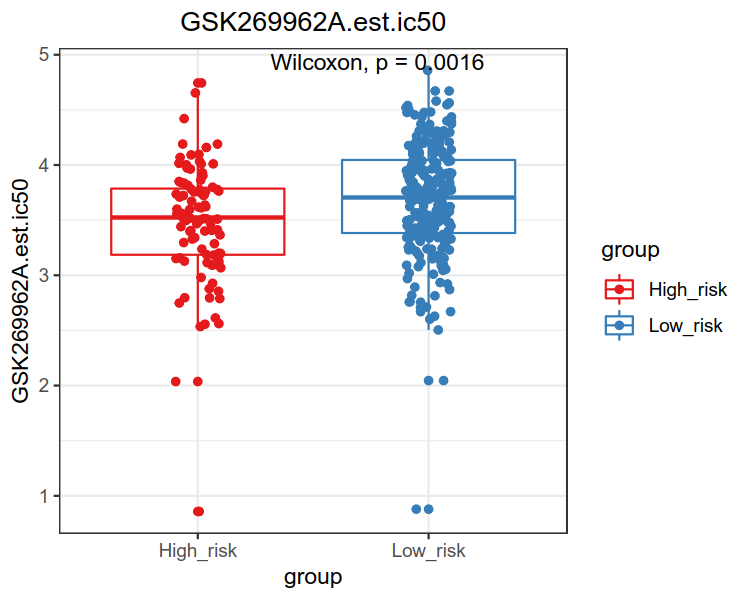

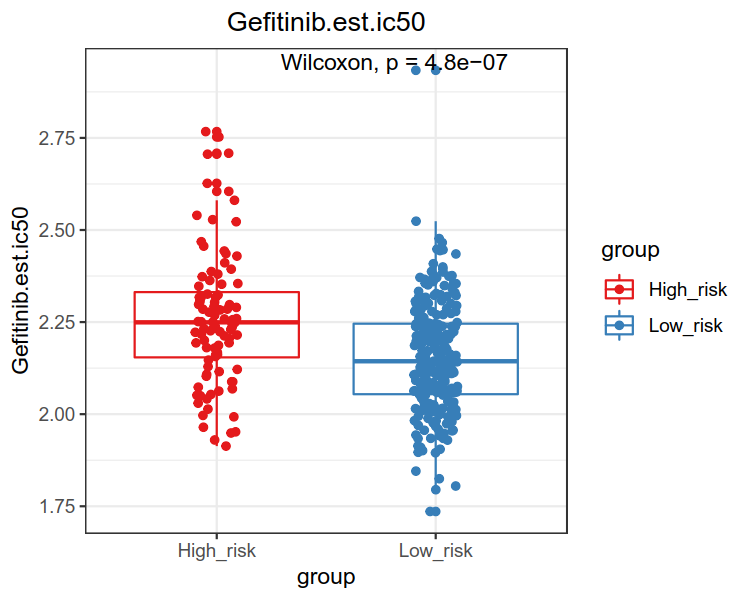

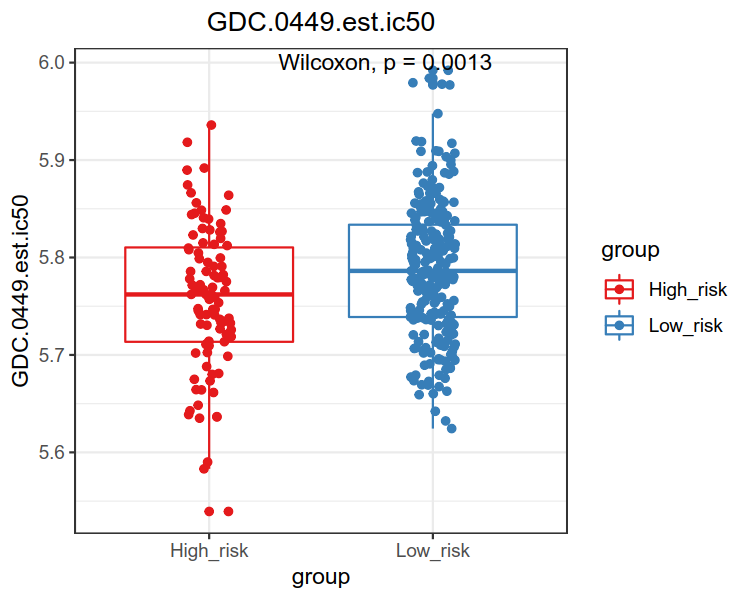

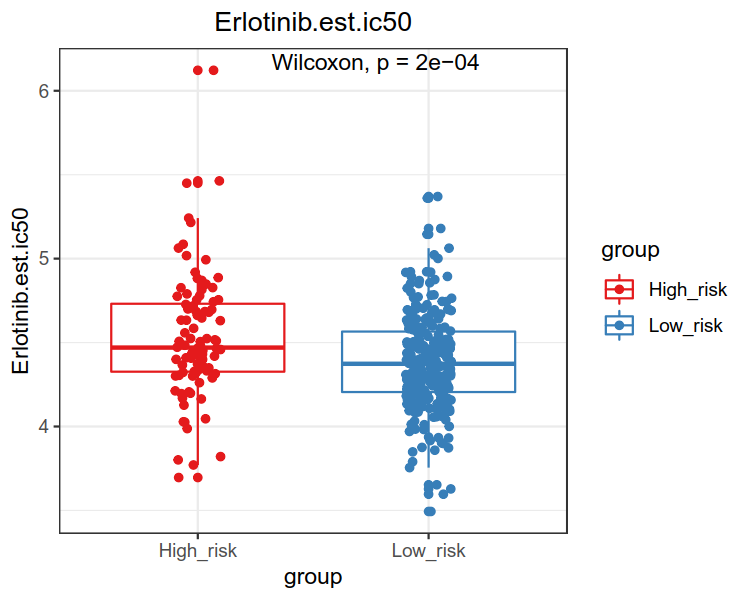

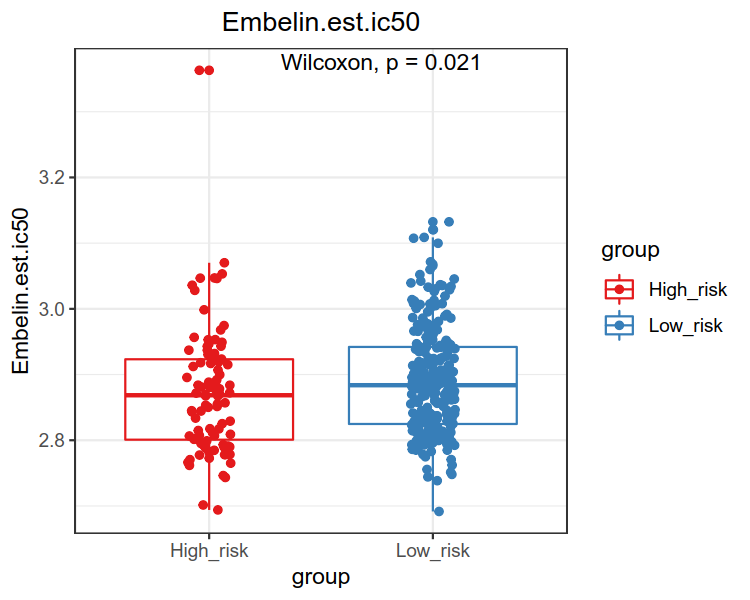

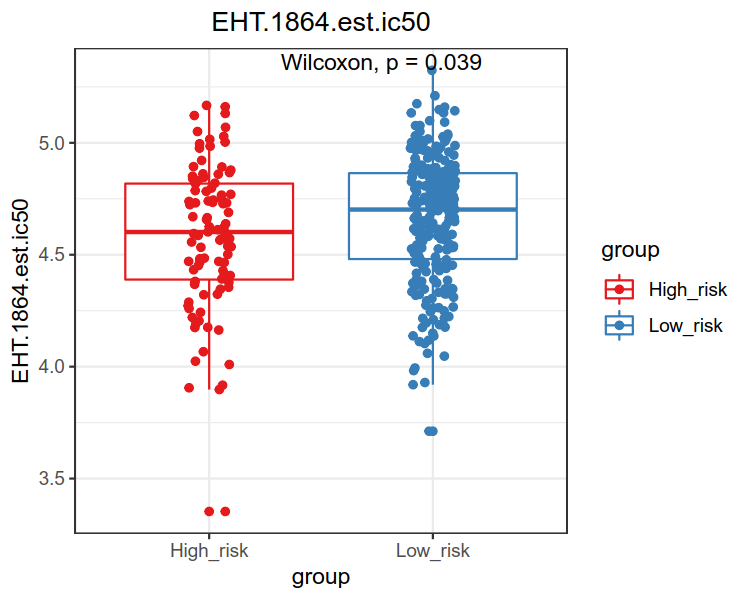

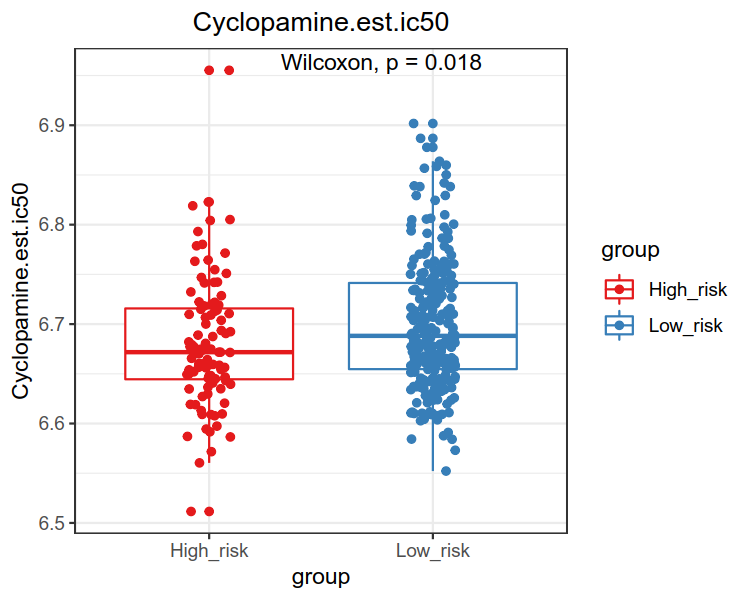

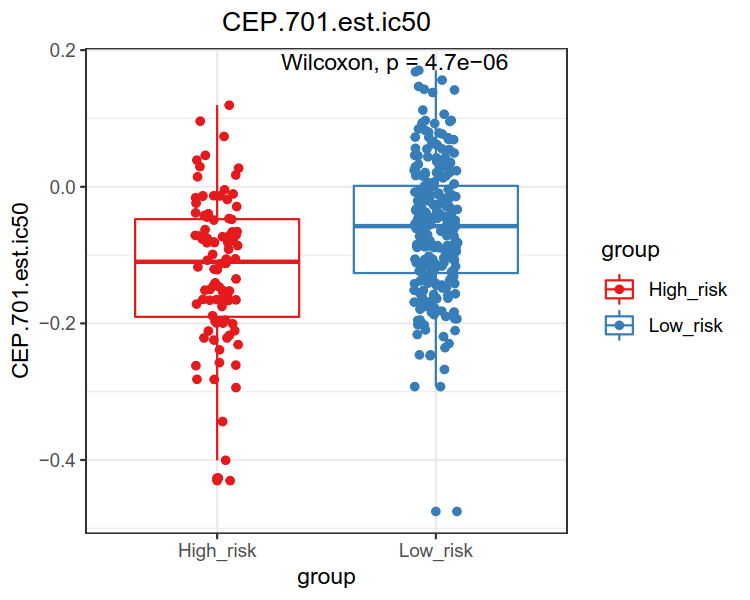

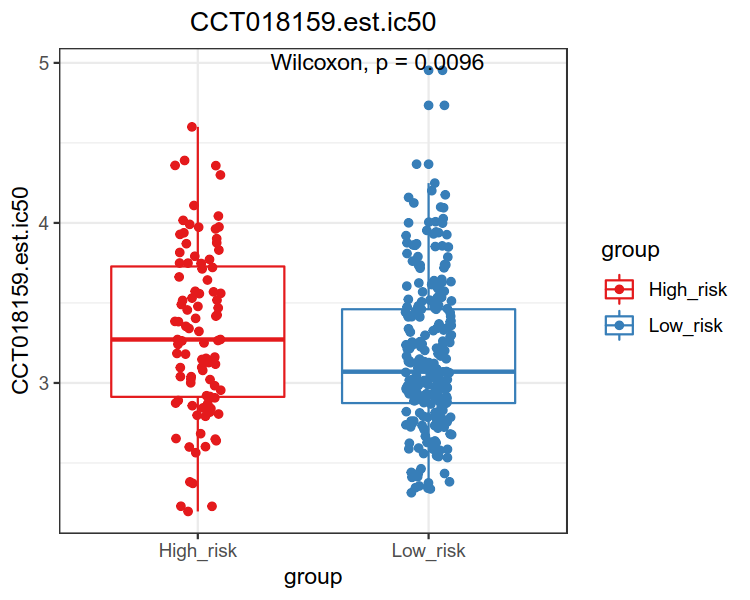

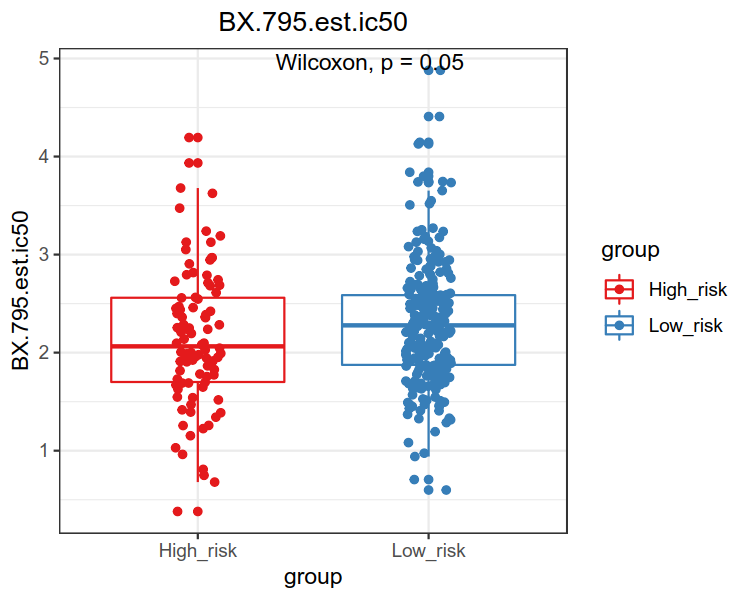

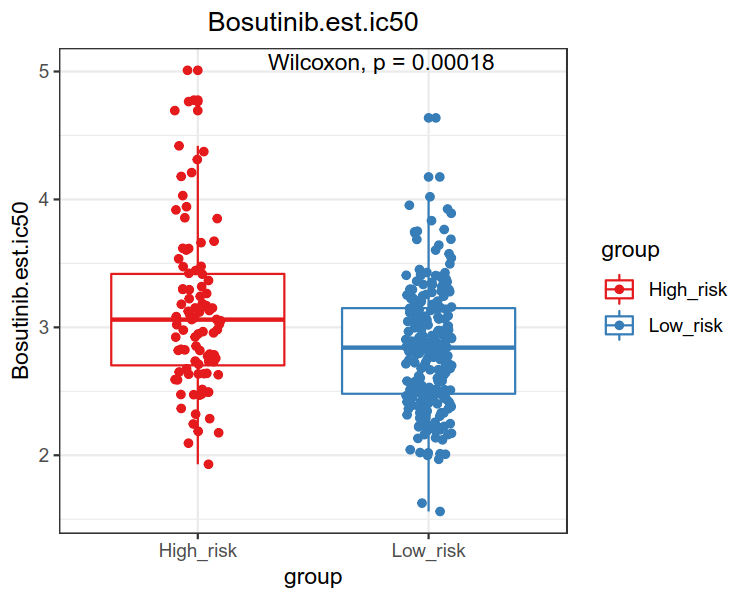

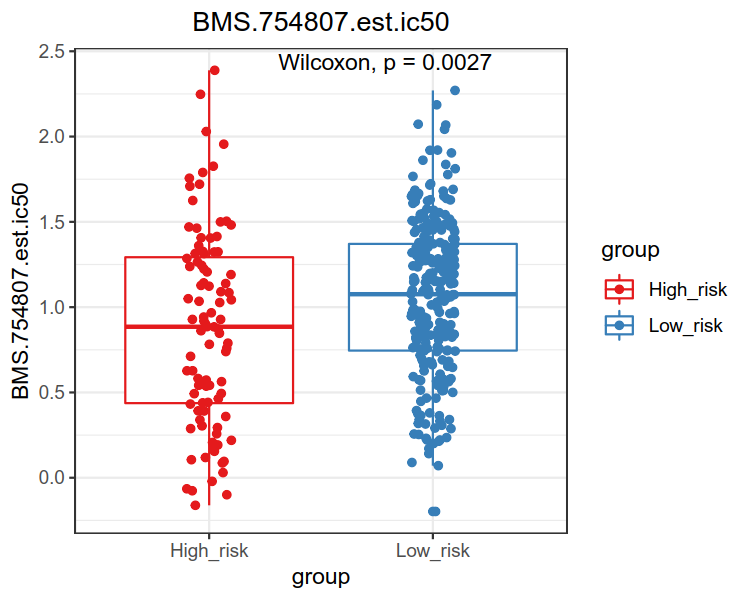

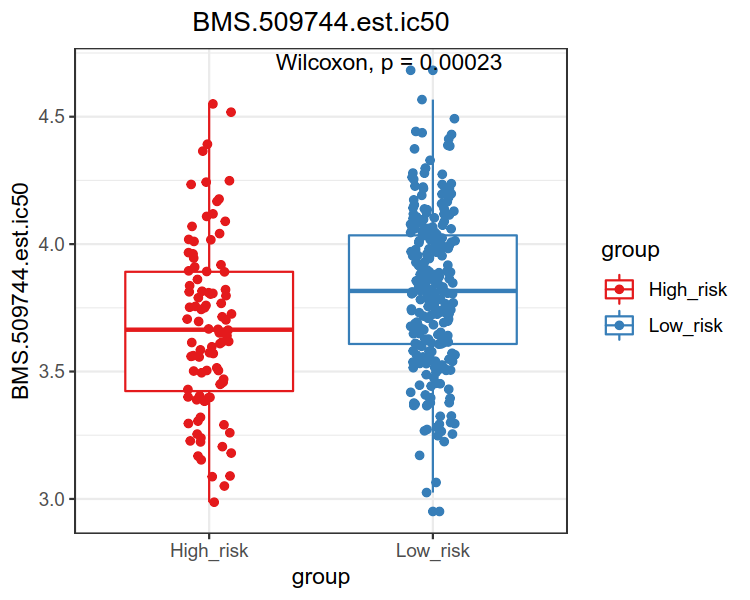

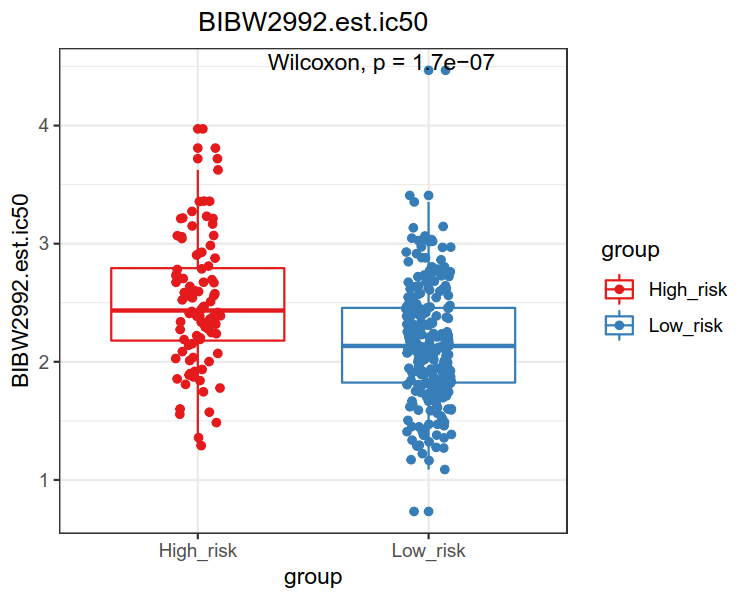

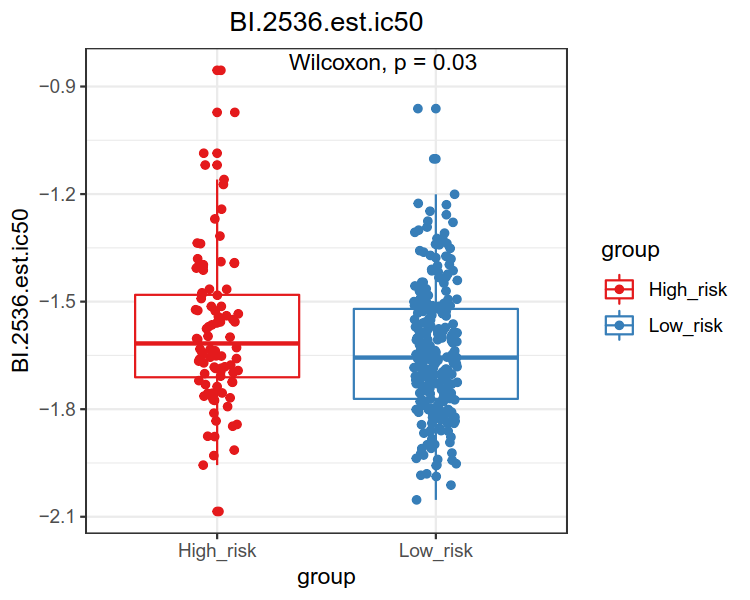

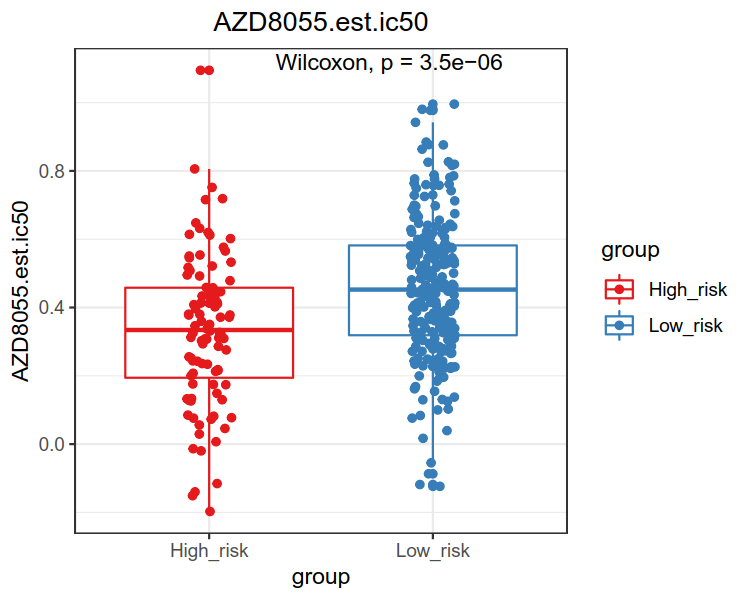

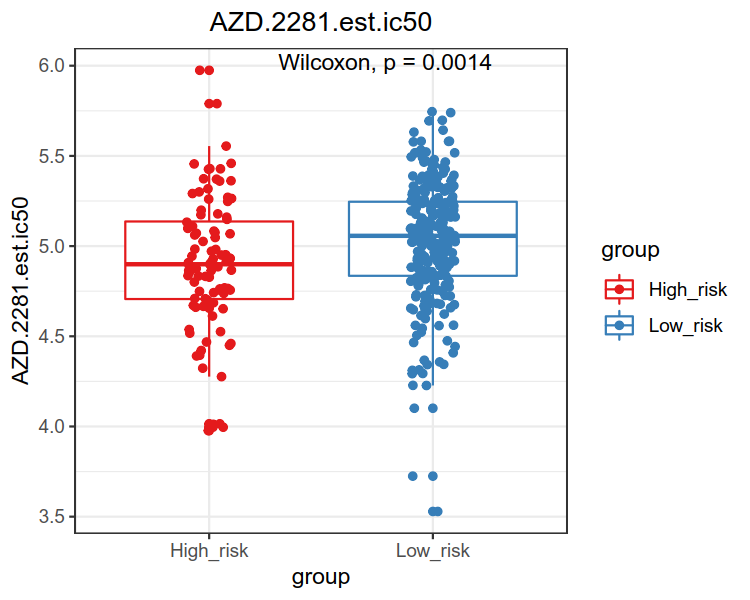

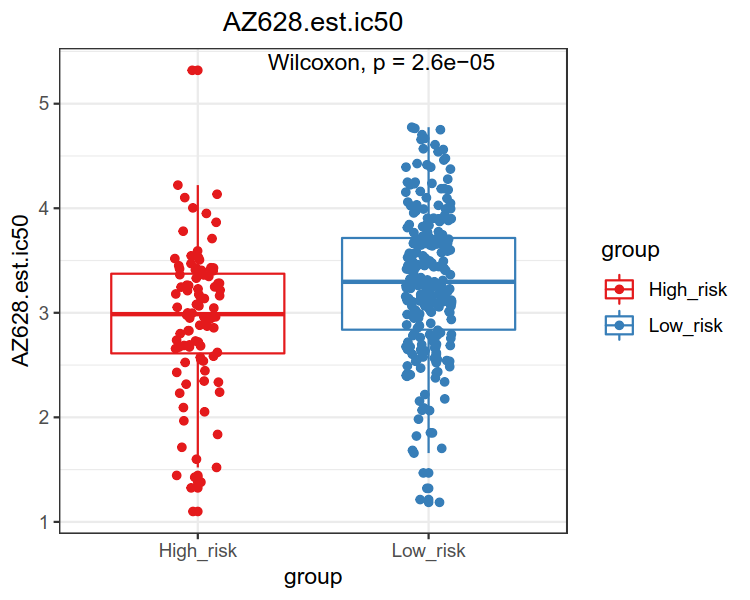

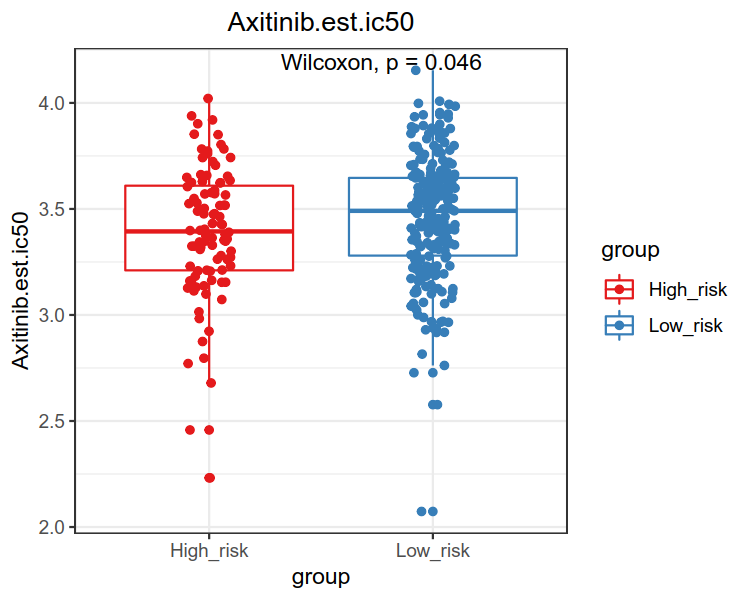

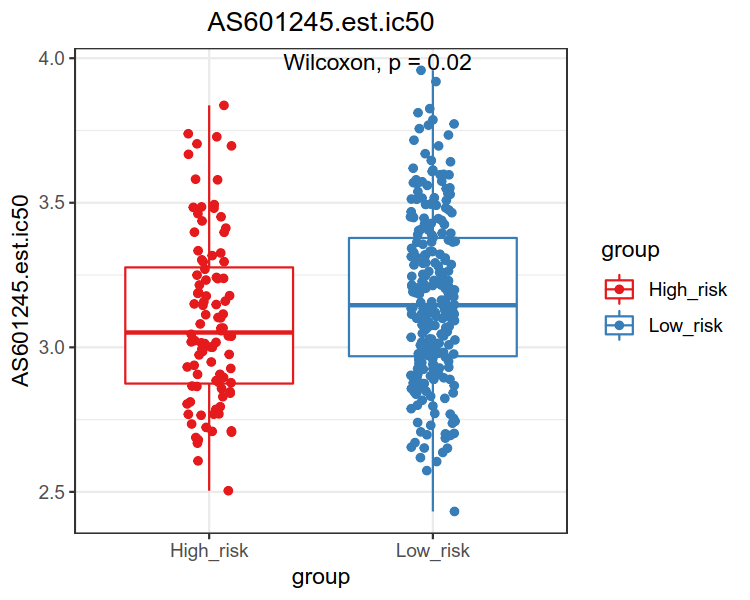

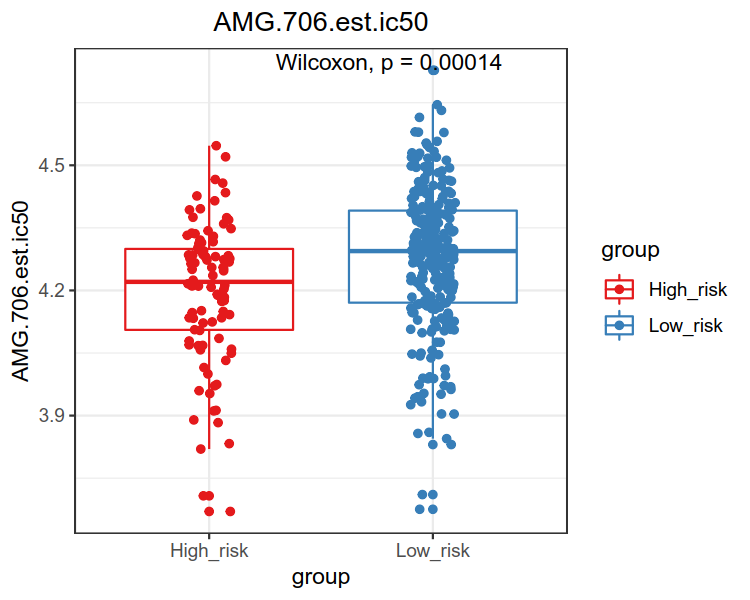

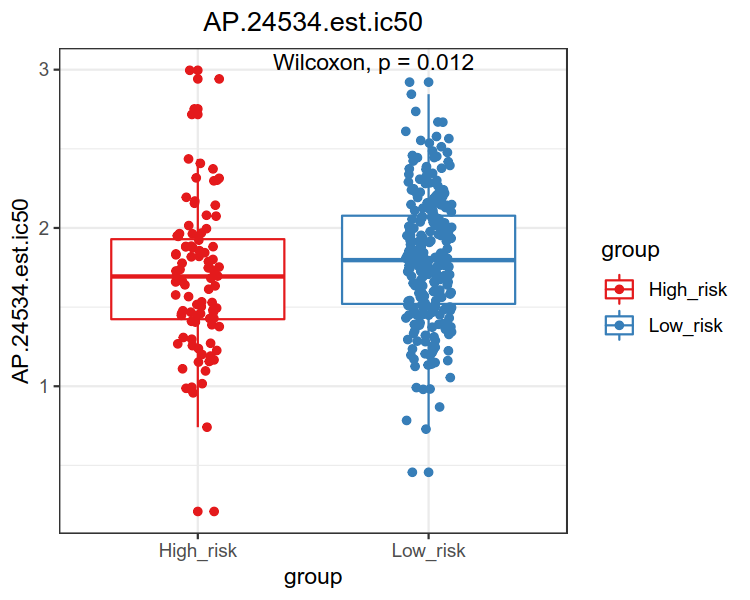

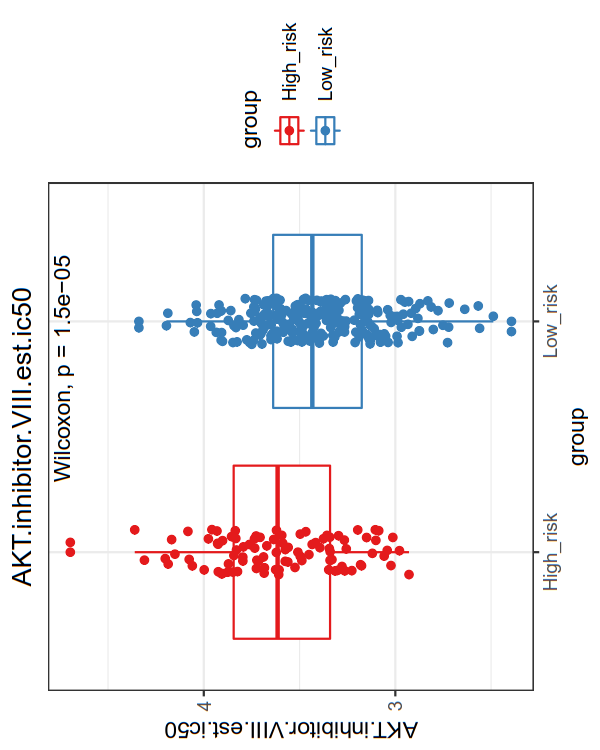

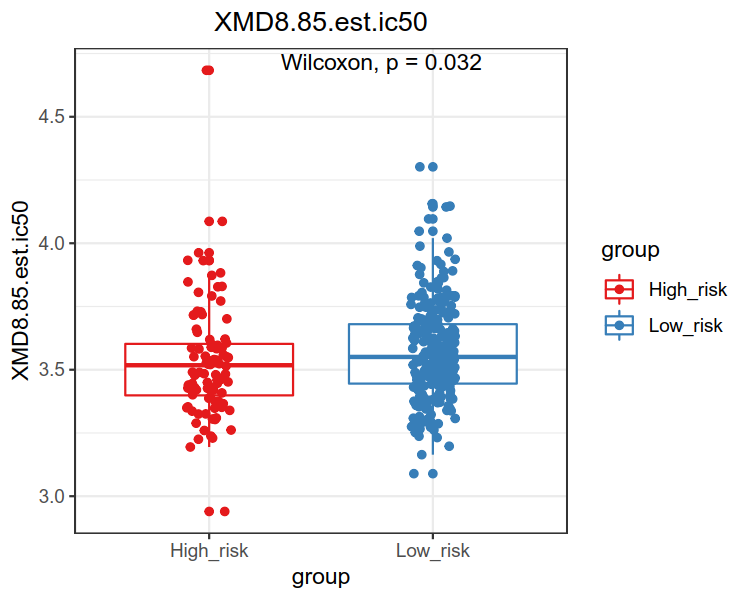

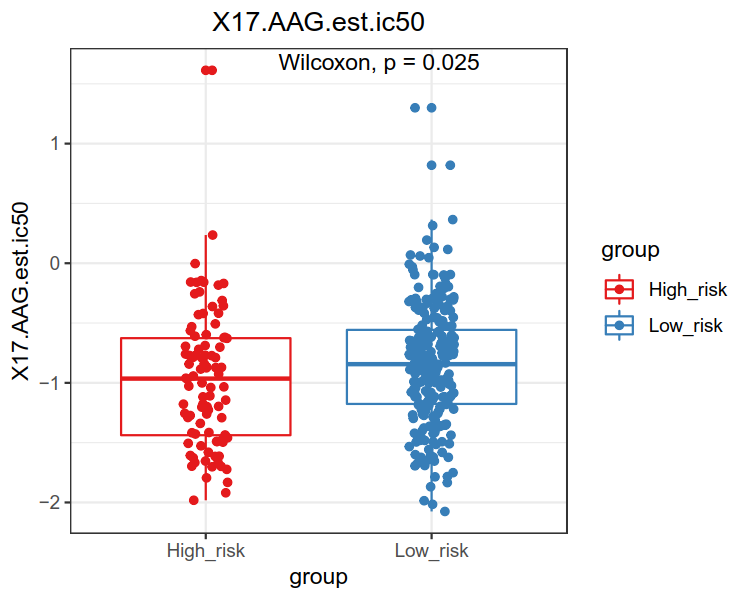

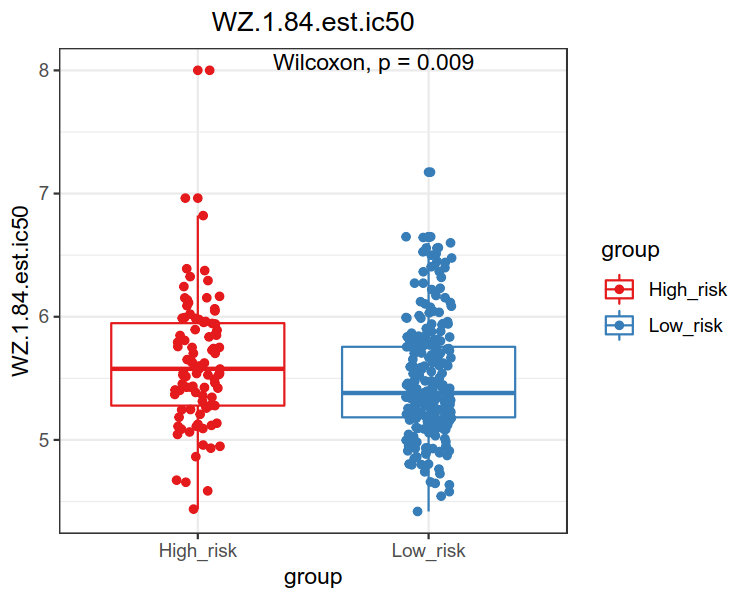

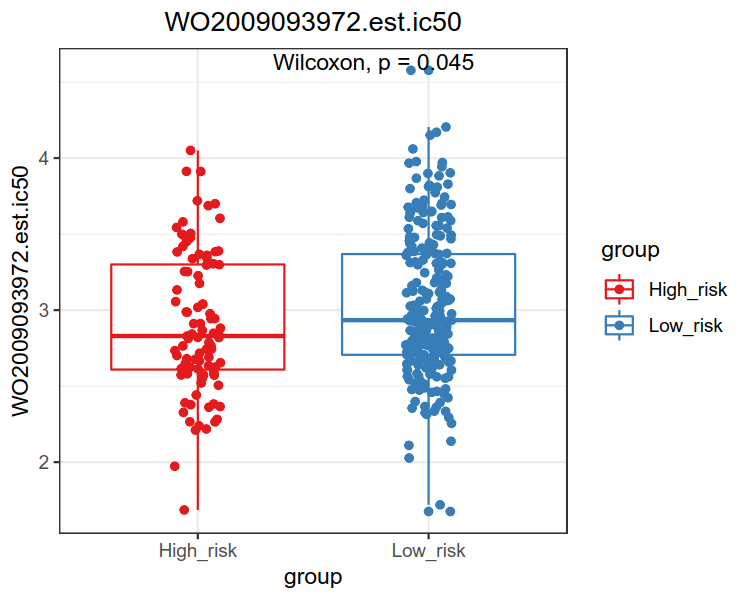

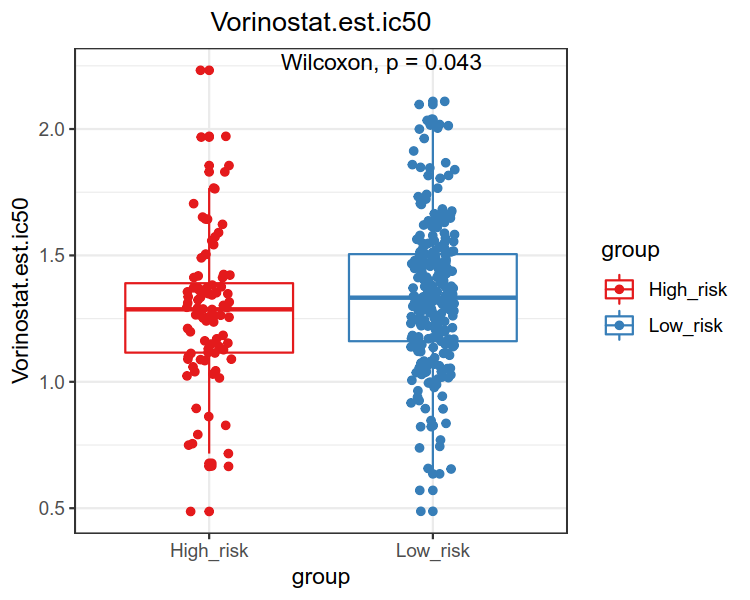

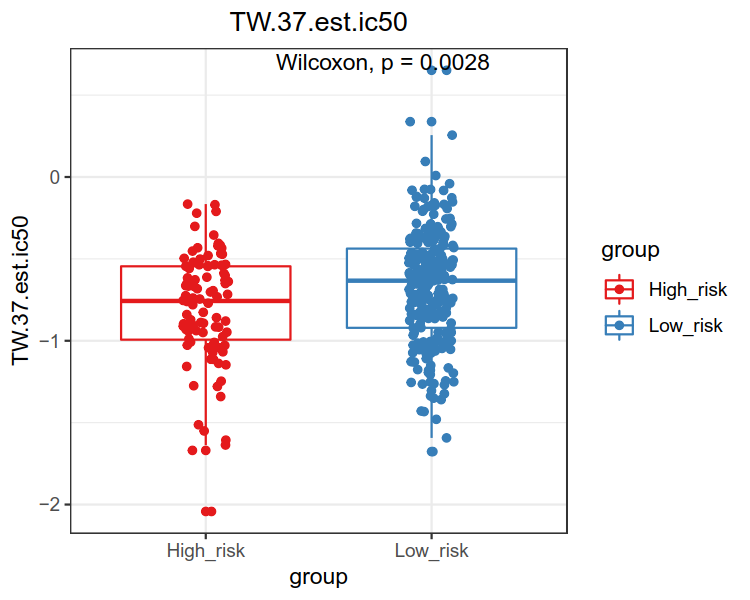

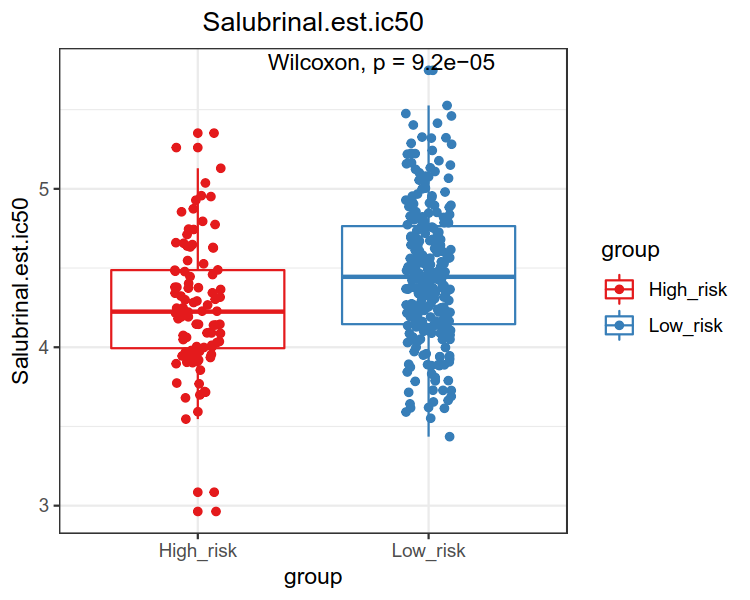

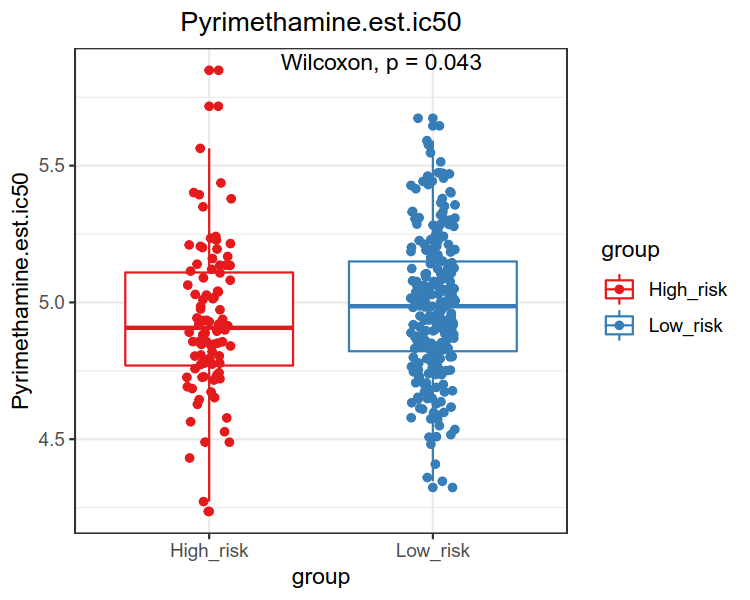

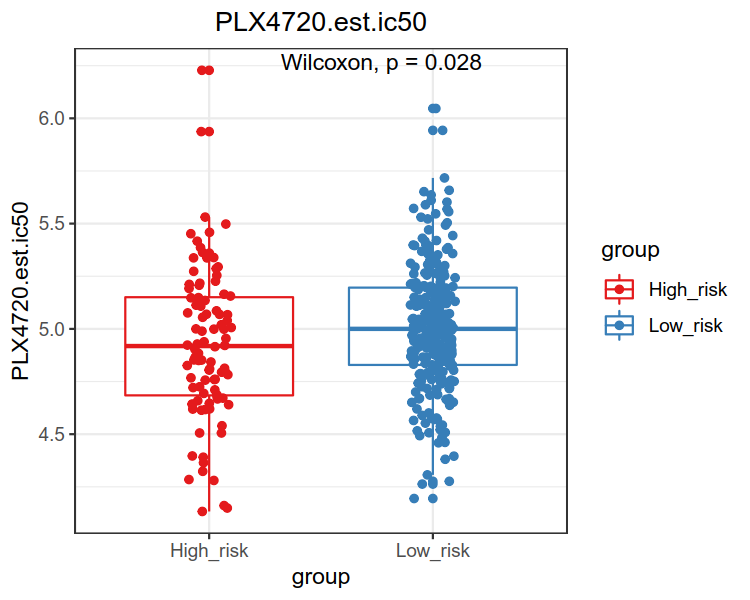

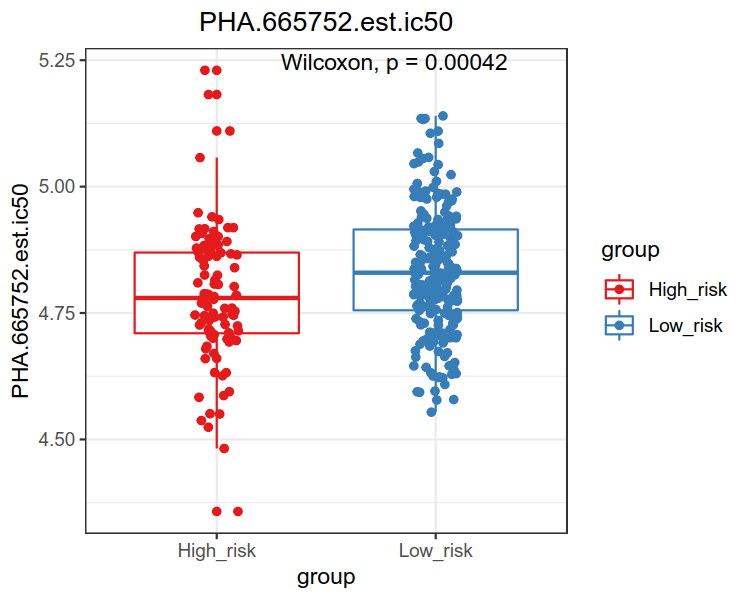

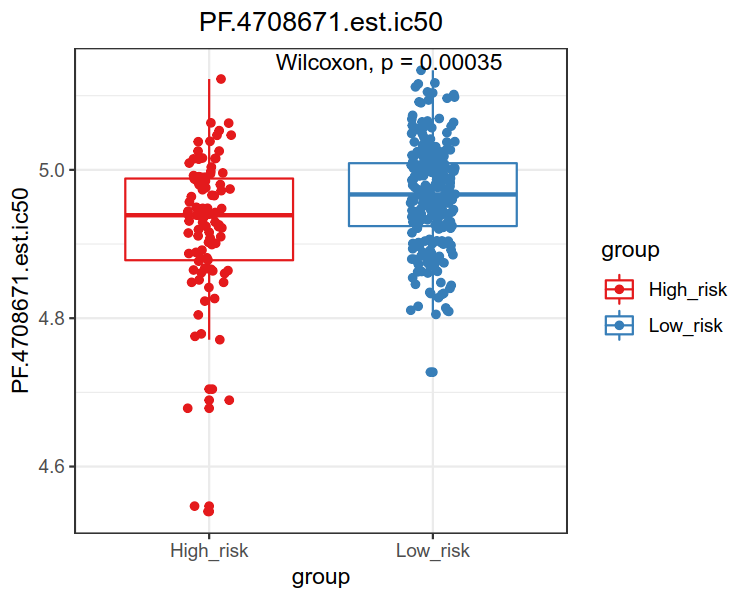

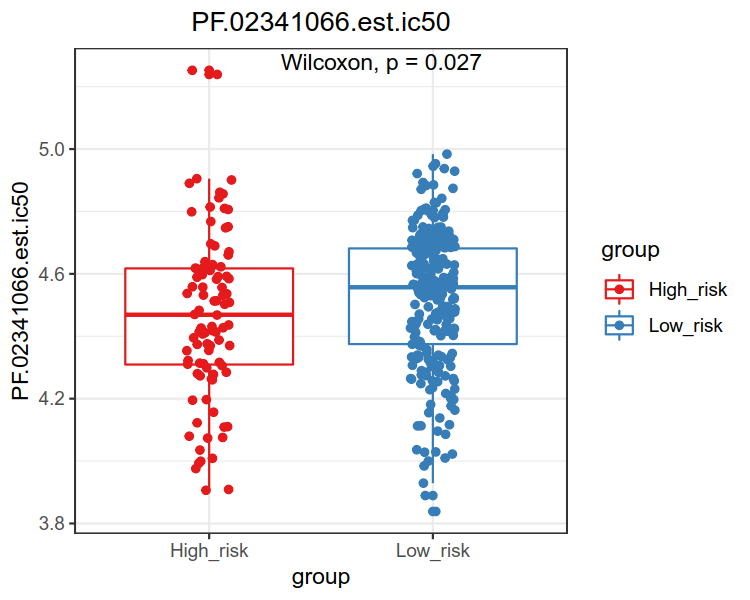

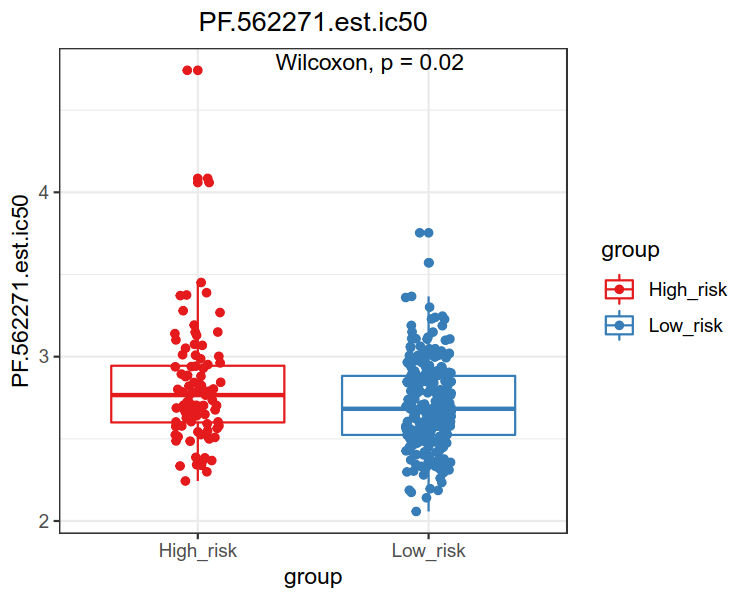

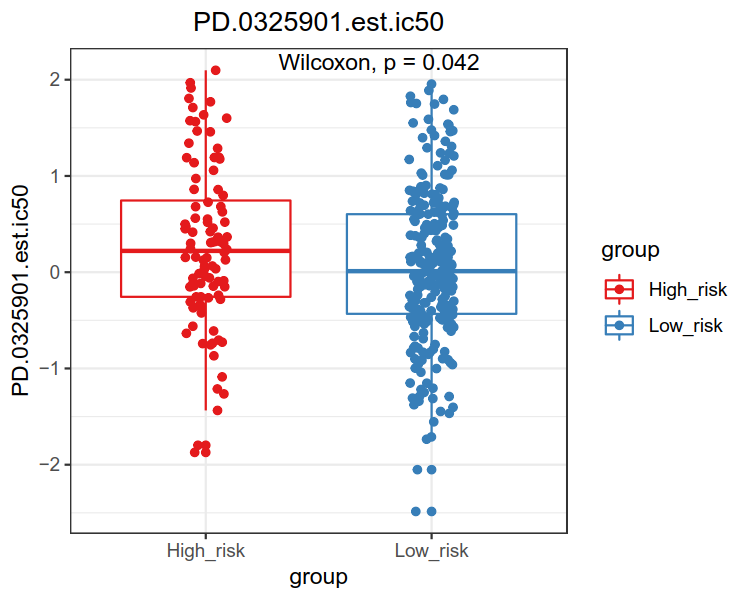
**

**Supplementary Figure 1. Analysis of chemotherapy response of 53 drugs**

**Supplementary Table 4. Differential results of drugs in high and low-risk groups**

| gene | normalMean | TumorMean | logFC | pValue | fdr |
| --- | --- | --- | --- | --- | --- |
| A.443654.est.ic50 | -0.6698 | -0.56585 | NaN | 0.025497 | 0.092595 |
| A.770041.est.ic50 | 3.692578 | 3.575017 | -0.04668 | 0.149742 | 0.303887 |
| ABT.263.est.ic50 | 2.957401 | 3.056363 | 0.047486 | 0.125909 | 0.271491 |
| ABT.888.est.ic50 | 5.451182 | 5.455288 | 0.001086 | 0.894027 | 0.938582 |
| AG.014699.est.ic50 | 4.189089 | 4.085455 | -0.03614 | 0.029484 | 0.094908 |
| AICAR.est.ic50 | 8.222668 | 8.363535 | 0.024506 | 0.059982 | 0.149809 |
| AKT.inhibitor.VIII.est.ic50 | 3.408881 | 3.606125 | 0.081151 | 1.53E-05 | 0.000265 |
| AMG.706.est.ic50 | 4.274578 | 4.197274 | -0.02633 | 0.000142 | 0.001787 |
| AP.24534.est.ic50 | 1.806414 | 1.689446 | -0.09658 | 0.012412 | 0.057096 |
| AS601245.est.ic50 | 3.171115 | 3.095172 | -0.03497 | 0.020011 | 0.079399 |
| ATRA.est.ic50 | 5.37923 | 5.378221 | -0.00027 | 0.922181 | 0.949708 |
| AUY922.est.ic50 | -2.83344 | -2.84499 | NaN | 0.242287 | 0.412786 |
| Axitinib.est.ic50 | 3.46312 | 3.39053 | -0.03056 | 0.0457 | 0.121281 |
| AZ628.est.ic50 | 3.289441 | 2.935956 | -0.16401 | 2.57E-05 | 0.000394 |
| AZD.0530.est.ic50 | 3.844706 | 3.851484 | 0.002541 | 0.640412 | 0.797241 |
| AZD.2281.est.ic50 | 5.024007 | 4.898192 | -0.03659 | 0.001438 | 0.010002 |
| AZD6244.est.ic50 | 3.077453 | 3.109625 | 0.015004 | 0.301549 | 0.489573 |
| AZD6482.est.ic50 | 3.447929 | 3.499559 | 0.021443 | 0.947603 | 0.965527 |
| AZD7762.est.ic50 | 0.008819 | -0.07546 | NaN | 0.183411 | 0.346723 |
| AZD8055.est.ic50 | 0.455407 | 0.332641 | -0.45319 | 3.49E-06 | 0.00012 |
| BAY.61.3606.est.ic50 | 2.641755 | 2.651795 | 0.005473 | 0.705195 | 0.836962 |
| Bexarotene.est.ic50 | 4.741943 | 4.731335 | -0.00323 | 0.655735 | 0.801854 |
| BI.2536.est.ic50 | -1.6391 | -1.58345 | NaN | 0.029573 | 0.094908 |
| BIBW2992.est.ic50 | 2.15656 | 2.495209 | 0.210429 | 1.69E-07 | 2.33E-05 |
| Bicalutamide.est.ic50 | 4.442506 | 4.453584 | 0.003593 | 0.408188 | 0.599255 |
| BI.D1870.est.ic50 | 2.571703 | 2.609526 | 0.021064 | 0.431383 | 0.61372 |
| BIRB.0796.est.ic50 | 5.897064 | 5.897699 | 0.000155 | 0.758637 | 0.856214 |
| Bleomycin.est.ic50 | 1.615643 | 1.563867 | -0.04699 | 0.253431 | 0.423007 |
| BMS.509744.est.ic50 | 3.813379 | 3.673014 | -0.05411 | 0.000226 | 0.002229 |
| BMS.536924.est.ic50 | 2.521346 | 2.478584 | -0.02468 | 0.210938 | 0.376076 |
| BMS.708163.est.ic50 | 4.941549 | 4.935633 | -0.00173 | 0.818775 | 0.891682 |
| BMS.754807.est.ic50 | 1.055403 | 0.874419 | -0.2714 | 0.002701 | 0.016081 |
| Bortezomib.est.ic50 | -5.31955 | -5.42573 | NaN | 0.123006 | 0.270073 |
| Bosutinib.est.ic50 | 2.835707 | 3.126223 | 0.140712 | 0.000177 | 0.002038 |
| Bryostatin.1.est.ic50 | -1.24904 | -1.24577 | NaN | 0.827067 | 0.891682 |
| BX.795.est.ic50 | 2.298445 | 2.144584 | -0.09996 | 0.049552 | 0.129022 |
| Camptothecin.est.ic50 | -3.89131 | -3.8614 | NaN | 0.549468 | 0.70866 |
| CCT007093.est.ic50 | 5.596038 | 5.601218 | 0.001335 | 0.361589 | 0.560666 |
| CCT018159.est.ic50 | 3.169763 | 3.315375 | 0.064797 | 0.009592 | 0.045645 |
| CEP.701.est.ic50 | -0.0605 | -0.12071 | NaN | 4.69E-06 | 0.000129 |
| CGP.082996.est.ic50 | 4.236806 | 4.250591 | 0.004686 | 0.834455 | 0.892672 |
| CGP.60474.est.ic50 | -1.60518 | -1.55337 | NaN | 0.311066 | 0.499152 |
| CHIR.99021.est.ic50 | 4.781577 | 4.773824 | -0.00234 | 0.335419 | 0.532043 |
| CI.1040.est.ic50 | 2.630177 | 2.713925 | 0.04522 | 0.456039 | 0.629334 |
| Cisplatin.est.ic50 | 3.392941 | 3.482062 | 0.037406 | 0.214414 | 0.376076 |
| CMK.est.ic50 | 3.676055 | 3.673821 | -0.00088 | 0.595374 | 0.760756 |
| Cyclopamine.est.ic50 | 6.699511 | 6.680927 | -0.00401 | 0.018368 | 0.079399 |
| Cytarabine.est.ic50 | 0.736996 | 0.628222 | -0.23038 | 0.340193 | 0.533484 |
| Dasatinib.est.ic50 | 1.513883 | 1.407438 | -0.10518 | 0.381873 | 0.57281 |
| DMOG.est.ic50 | 6.744133 | 6.877107 | 0.028169 | 0.14511 | 0.298884 |
| Docetaxel.est.ic50 | -5.41106 | -5.42895 | NaN | 0.755035 | 0.856214 |
| Doxorubicin.est.ic50 | -1.89575 | -1.85983 | NaN | 0.40284 | 0.597763 |
| EHT.1864.est.ic50 | 4.663674 | 4.584366 | -0.02474 | 0.038974 | 0.117597 |
| Elesclomol.est.ic50 | -3.04459 | -3.05686 | NaN | 0.755035 | 0.856214 |
| Embelin.est.ic50 | 2.890704 | 2.87193 | -0.0094 | 0.02111 | 0.080921 |
| Epothilone.B.est.ic50 | -5.11707 | -5.04601 | NaN | 0.21529 | 0.376076 |
| Erlotinib.est.ic50 | 4.38721 | 4.534719 | 0.047709 | 0.000196 | 0.002077 |
| Etoposide.est.ic50 | 1.692875 | 1.782503 | 0.074428 | 0.280983 | 0.461615 |
| FH535.est.ic50 | 2.081007 | 2.033645 | -0.03321 | 0.140589 | 0.29396 |
| FTI.277.est.ic50 | 3.626721 | 3.677285 | 0.019975 | 0.193759 | 0.356517 |
| GDC.0449.est.ic50 | 5.788806 | 5.757899 | -0.00772 | 0.001292 | 0.009905 |
| GDC0941.est.ic50 | 2.239528 | 2.131293 | -0.07147 | 0.05222 | 0.133451 |
| Gefitinib.est.ic50 | 2.151796 | 2.258953 | 0.070112 | 4.83E-07 | 2.95E-05 |
| Gemcitabine.est.ic50 | -2.28524 | -2.39707 | NaN | 0.641259 | 0.797241 |
| GNF.2.est.ic50 | 4.492885 | 4.481821 | -0.00356 | 0.506894 | 0.679139 |
| GSK269962A.est.ic50 | 3.684027 | 3.482737 | -0.08106 | 0.001631 | 0.010399 |
| GSK.650394.est.ic50 | 4.073605 | 4.081115 | 0.002657 | 0.198264 | 0.360006 |
| GW.441756.est.ic50 | 4.258005 | 4.286061 | 0.009475 | 0.044557 | 0.121281 |
| GW843682X.est.ic50 | -2.23681 | -2.23663 | NaN | 0.897774 | 0.938582 |
| Imatinib.est.ic50 | 4.939189 | 4.913757 | -0.00745 | 0.060792 | 0.149809 |
| IPA.3.est.ic50 | 6.074256 | 5.962179 | -0.02687 | 0.001658 | 0.010399 |
| JNJ.26854165.est.ic50 | 2.911629 | 2.894975 | -0.00828 | 0.443261 | 0.624184 |
| JNK.9L.est.ic50 | -0.20112 | -0.12524 | NaN | 0.119312 | 0.269918 |
| JNK.Inhibitor.VIII.est.ic50 | 5.908319 | 5.905123 | -0.00078 | 0.793109 | 0.875592 |
| JW.7.52.1.est.ic50 | -1.53373 | -1.56926 | NaN | 0.65659 | 0.801854 |
| KIN001.135.est.ic50 | 5.967347 | 5.97649 | 0.002209 | 0.427235 | 0.61372 |
| KU.55933.est.ic50 | 5.161185 | 5.14462 | -0.00464 | 0.018663 | 0.079399 |
| Lapatinib.est.ic50 | 4.035229 | 4.190351 | 0.054421 | 0.003798 | 0.020962 |
| Lenalidomide.est.ic50 | 5.470367 | 5.481639 | 0.00297 | 0.074837 | 0.181184 |
| LFM.A13.est.ic50 | 6.191805 | 6.213934 | 0.005147 | 0.479996 | 0.649406 |
| Metformin.est.ic50 | 10.5268 | 10.64693 | 0.01637 | 0.009592 | 0.045645 |
| Methotrexate.est.ic50 | 1.25501 | 1.284174 | 0.033143 | 0.958921 | 0.965527 |
| MG.132.est.ic50 | 1.374552 | 1.238214 | -0.1507 | 0.02844 | 0.094908 |
| Midostaurin.est.ic50 | 0.595285 | 0.353501 | -0.75187 | 1.11E-05 | 0.000255 |
| Mitomycin.C.est.ic50 | -0.94363 | -0.8949 | NaN | 0.450335 | 0.627739 |
| MK.2206.est.ic50 | 2.864048 | 2.850667 | -0.00676 | 0.020138 | 0.079399 |
| MS.275.est.ic50 | 0.969582 | 0.919885 | -0.07591 | 0.254417 | 0.423007 |
| Nilotinib.est.ic50 | 4.405648 | 4.407083 | 0.00047 | 0.763147 | 0.856214 |
| NSC.87877.est.ic50 | 6.739081 | 6.745601 | 0.001395 | 0.466844 | 0.637867 |
| NU.7441.est.ic50 | 3.564979 | 3.526494 | -0.01566 | 6.41E-07 | 2.95E-05 |
| Nutlin.3a.est.ic50 | 4.863327 | 4.804202 | -0.01765 | 0.153459 | 0.306918 |
| NVP.BEZ235.est.ic50 | -2.30491 | -2.47606 | NaN | 0.001175 | 0.009534 |
| NVP.TAE684.est.ic50 | 1.587906 | 1.567611 | -0.01856 | 0.096302 | 0.225248 |
| Obatoclax.Mesylate.est.ic50 | -0.94545 | -1.02293 | NaN | 0.370379 | 0.567914 |
| OSI.906.est.ic50 | 3.60808 | 3.536187 | -0.02904 | 0.102125 | 0.234888 |
| PAC.1.est.ic50 | 3.812074 | 3.798114 | -0.00529 | 0.180311 | 0.345596 |
| Paclitaxel.est.ic50 | -2.91108 | -2.78094 | NaN | 0.039199 | 0.117597 |
| Parthenolide.est.ic50 | 4.991789 | 4.908446 | -0.02429 | 0.006637 | 0.035228 |
| Pazopanib.est.ic50 | 4.499849 | 4.34784 | -0.04958 | 1.45E-05 | 0.000265 |
| PD.0325901.est.ic50 | 0.060246 | 0.268017 | 2.153378 | 0.041509 | 0.121281 |
| PD.0332991.est.ic50 | 2.481343 | 2.466746 | -0.00851 | 0.825223 | 0.891682 |
| PD.173074.est.ic50 | 4.224527 | 4.152507 | -0.02481 | 0.001449 | 0.010002 |
| PF.02341066.est.ic50 | 4.521096 | 4.467453 | -0.01722 | 0.027345 | 0.094908 |
| PF.4708671.est.ic50 | 4.964344 | 4.922669 | -0.01216 | 0.00035 | 0.003218 |
| PF.562271.est.ic50 | 2.705088 | 2.817535 | 0.058758 | 0.019635 | 0.079399 |
| PHA.665752.est.ic50 | 4.83379 | 4.781612 | -0.01566 | 0.000424 | 0.00366 |
| PLX4720.est.ic50 | 5.01133 | 4.92681 | -0.02454 | 0.027595 | 0.094908 |
| Pyrimethamine.est.ic50 | 4.987838 | 4.933001 | -0.01595 | 0.042828 | 0.121281 |
| QS11.est.ic50 | 3.887642 | 3.869288 | -0.00683 | 0.777633 | 0.86543 |
| Rapamycin.est.ic50 | -0.00802 | 0.012394 | NaN | 0.678125 | 0.820888 |
| RDEA119.est.ic50 | 2.28094 | 2.29238 | 0.007217 | 0.965527 | 0.965527 |
| RO.3306.est.ic50 | 4.672802 | 4.689365 | 0.005105 | 0.63028 | 0.797241 |
| Roscovitine.est.ic50 | 4.930393 | 4.924896 | -0.00161 | 0.159693 | 0.314822 |
| Salubrinal.est.ic50 | 4.464475 | 4.256839 | -0.06871 | 9.17E-05 | 0.001265 |
| SB.216763.est.ic50 | 5.607428 | 5.589833 | -0.00453 | 0.521403 | 0.685272 |
| SB590885.est.ic50 | 5.105312 | 5.105186 | ######## | 0.756835 | 0.856214 |
| Shikonin.est.ic50 | 0.242308 | 0.302912 | 0.322055 | 0.376738 | 0.571317 |
| SL.0101.1.est.ic50 | 5.695449 | 5.686122 | -0.00236 | 0.840931 | 0.89268 |
| Sorafenib.est.ic50 | 3.907399 | 3.879061 | -0.0105 | 0.127972 | 0.271695 |
| S.Trityl.L.cysteine.est.ic50 | 1.661117 | 1.679579 | 0.015946 | 0.527575 | 0.686843 |
| Sunitinib.est.ic50 | 3.760732 | 3.750607 | -0.00389 | 0.179542 | 0.345596 |
| Temsirolimus.est.ic50 | -0.99051 | -0.98393 | NaN | 0.957977 | 0.965527 |
| Thapsigargin.est.ic50 | -4.2909 | -4.43246 | NaN | 0.123294 | 0.270073 |
| Tipifarnib.est.ic50 | 2.157311 | 2.188866 | 0.020949 | 0.706075 | 0.836962 |
| TW.37.est.ic50 | -0.6701 | -0.8087 | NaN | 0.002797 | 0.016081 |
| Vinblastine.est.ic50 | -4.0836 | -4.00408 | NaN | 0.240385 | 0.412786 |
| Vinorelbine.est.ic50 | -3.8628 | -3.74472 | NaN | 0.079189 | 0.188414 |
| Vorinostat.est.ic50 | 1.344528 | 1.277574 | -0.07369 | 0.043316 | 0.121281 |
| VX.680.est.ic50 | 2.658604 | 2.661532 | 0.001588 | 0.729972 | 0.853697 |
| VX.702.est.ic50 | 4.457097 | 4.418776 | -0.01246 | 0.192544 | 0.356517 |
| WH.4.023.est.ic50 | 3.820538 | 3.784062 | -0.01384 | 0.908089 | 0.942228 |
| WO2009093972.est.ic50 | 3.021886 | 2.909469 | -0.05469 | 0.044936 | 0.121281 |
| WZ.1.84.est.ic50 | 5.490427 | 5.623737 | 0.034611 | 0.009046 | 0.045645 |
| X17.AAG.est.ic50 | -0.85059 | -0.97546 | NaN | 0.024804 | 0.092512 |
| X681640.est.ic50 | 2.551596 | 2.590854 | 0.022028 | 0.709598 | 0.836962 |
| XMD8.85.est.ic50 | 3.572458 | 3.532693 | -0.01615 | 0.032335 | 0.101415 |
| Z.LLNle.CHO.est.ic50 | 2.743677 | 2.72185 | -0.01152 | 0.421741 | 0.612634 |
| ZM.447439.est.ic50 | 2.833012 | 2.808338 | -0.01262 | 0.521403 | 0.685272 |
